# Supplementary material for: Topological stress is responsible for the detrimental outcomes of head-on replication-transcription conflicts
Source: Cell Rep. Author manuscript; Available in PMC 2021 Mar 23. (PMC7986047; doi:10.1016/j.celrep.2021.108797)
Supplement: 1 [file NIHMS1679665-supplement-1.pdf]

Cell Reports, Volume *34*

Supplemental information

Topological stress is responsible for the detrimental outcomes of head-on replication-transcription conflicts

Kevin S. Lang and Houra Merrikh

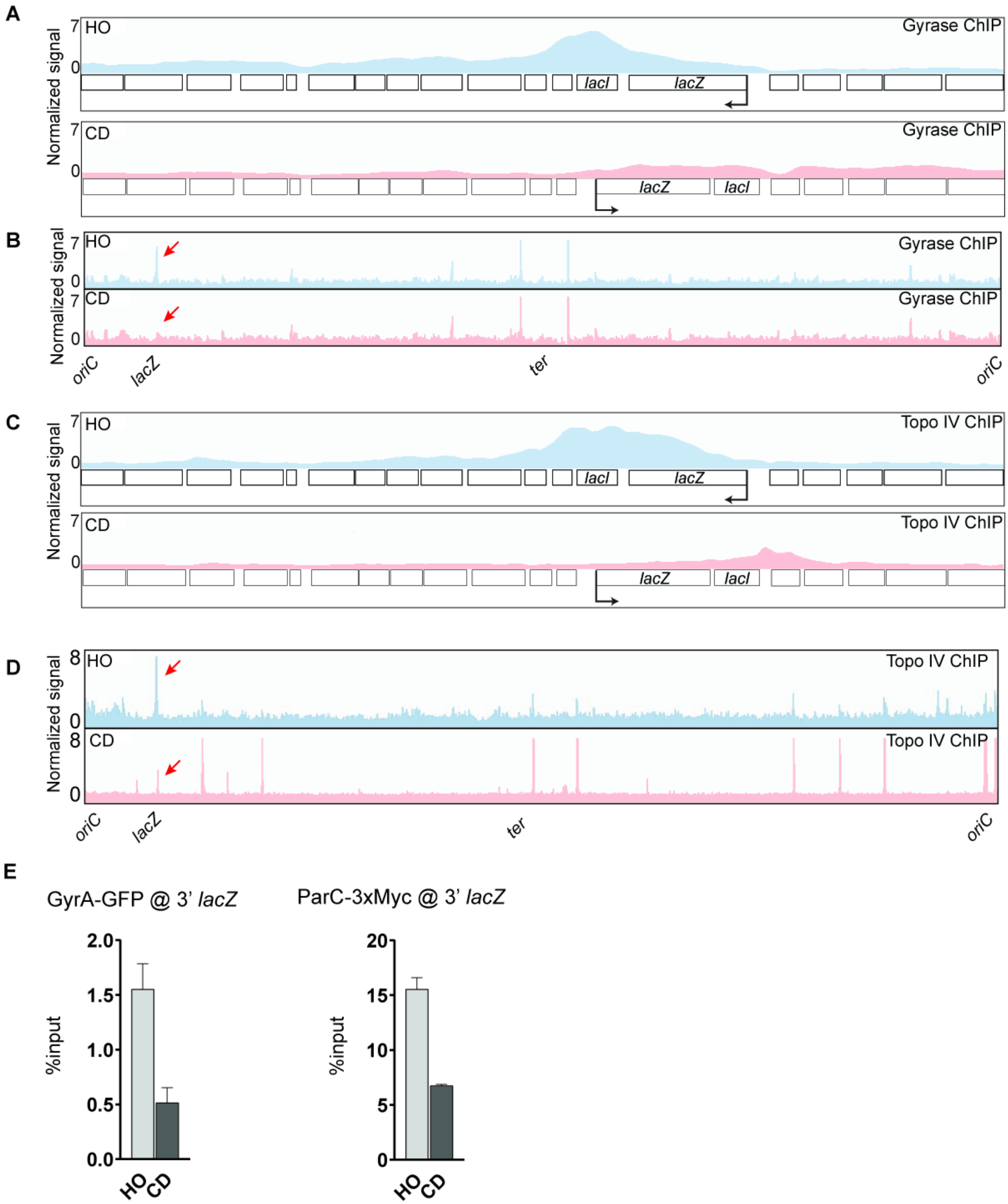

Supplementary Figure 1. Association of type II topoisomerases with head-on genes is dependent on transcription. Related to Figure 1. Replicate ChIP-Seq profiles of **(A)** gyrase and **(C)** Topo IV at the engineered conflict locus and **(B)** and **(D)** genome-wide in cells carrying either a head-on (HO, blue, strain HM3863 (gyrase), HM4074 (ParC)) or co-directional (CD, red, strain HM3864 (gyrase), HM4075 (ParC)) *lacZ* engineered conflict. The direction of DNA replication is left to right. Direction of transcription is indicated by the promoter arrow on *lacZ*. **(E)** ChIP-qPCR data from **Fig. 1C-D** presented as percent input. Bars represent the mean and standard error.

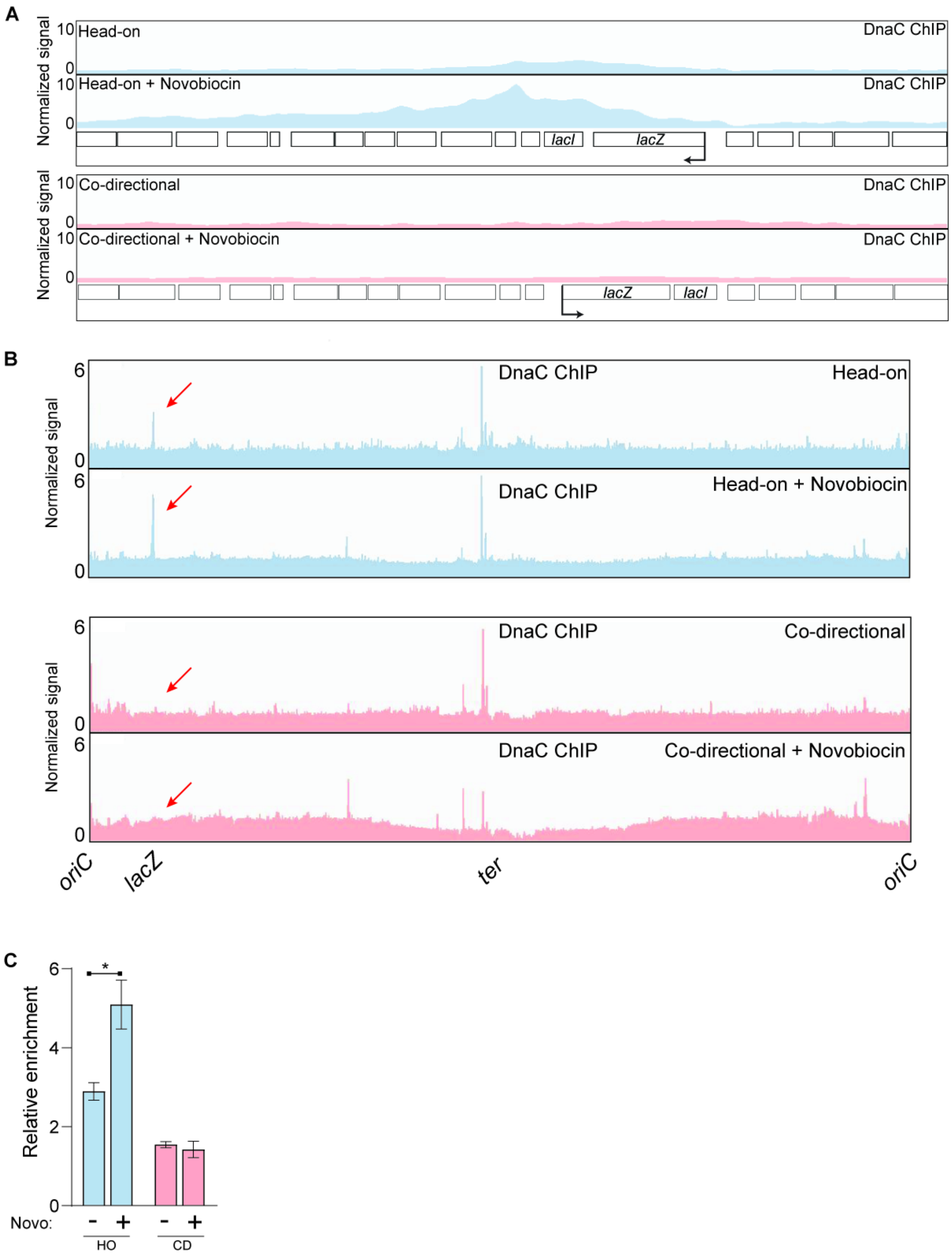

Supplementary Figure 2. Association of the replicative helicase at engineered conflict regions. Related to Figure 2. Replicate ChIP-Seq profiles at the engineered conflict locus **(A)** and genome-wide **b,** of DnaC in cells carrying either a head-on (HO, blue, strain HM1300) or co-directional (CD, red, strain HM1416) *lacZ*

engineered conflict, with and without novobiocin treatment (375 ng/mL). The direction of DNA replication is left to right. Direction of transcription is indicated by the promoter arrow on *lacZ*. Red arrow shows the location of the engineered conflict locus in the genome-wide plot. **c,** ChIP-qPCR analysis of the 3' region of *lacZ* with and without novobiocin treatment. The bars represent the mean and standard error of 4 biological replicates. (*) represents $p < 0.05$.

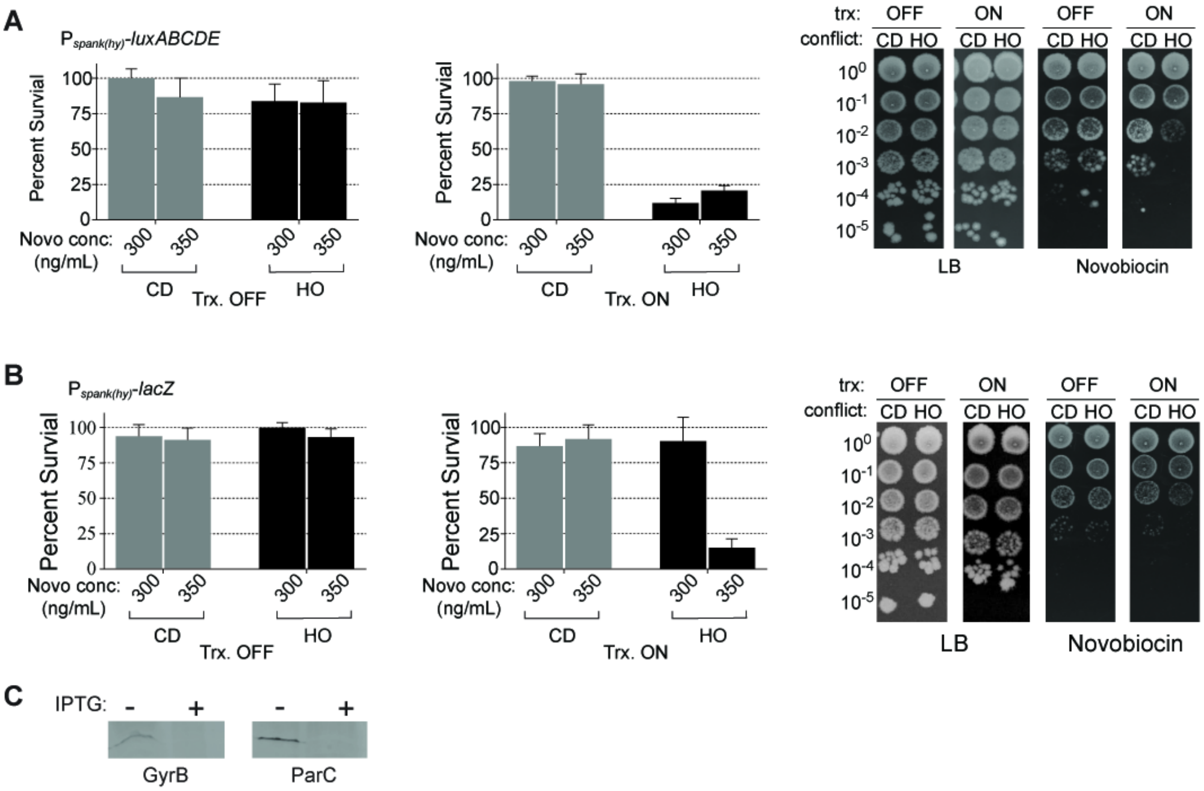

Supplementary Figure 3. Sensitivity of cells expressing a highly transcribed gene is independent of gene sequence and genomic location. Related to Figure 3. Novobiocin survival assays of cells expressing $P_{spank(hy)}$-*luxABCDE* **(A)** and $P_{spank(hy)}$-*lacZ* **(B)** at the *amyE* locus. Quantification is shown as percent survival (average, +/- standard deviation). Representative plates of the highest novobiocin concentration (350 ng/mL) are shown. **(C)** Slot blot analysis of depletions of GyrB and ParC using the degron systems.

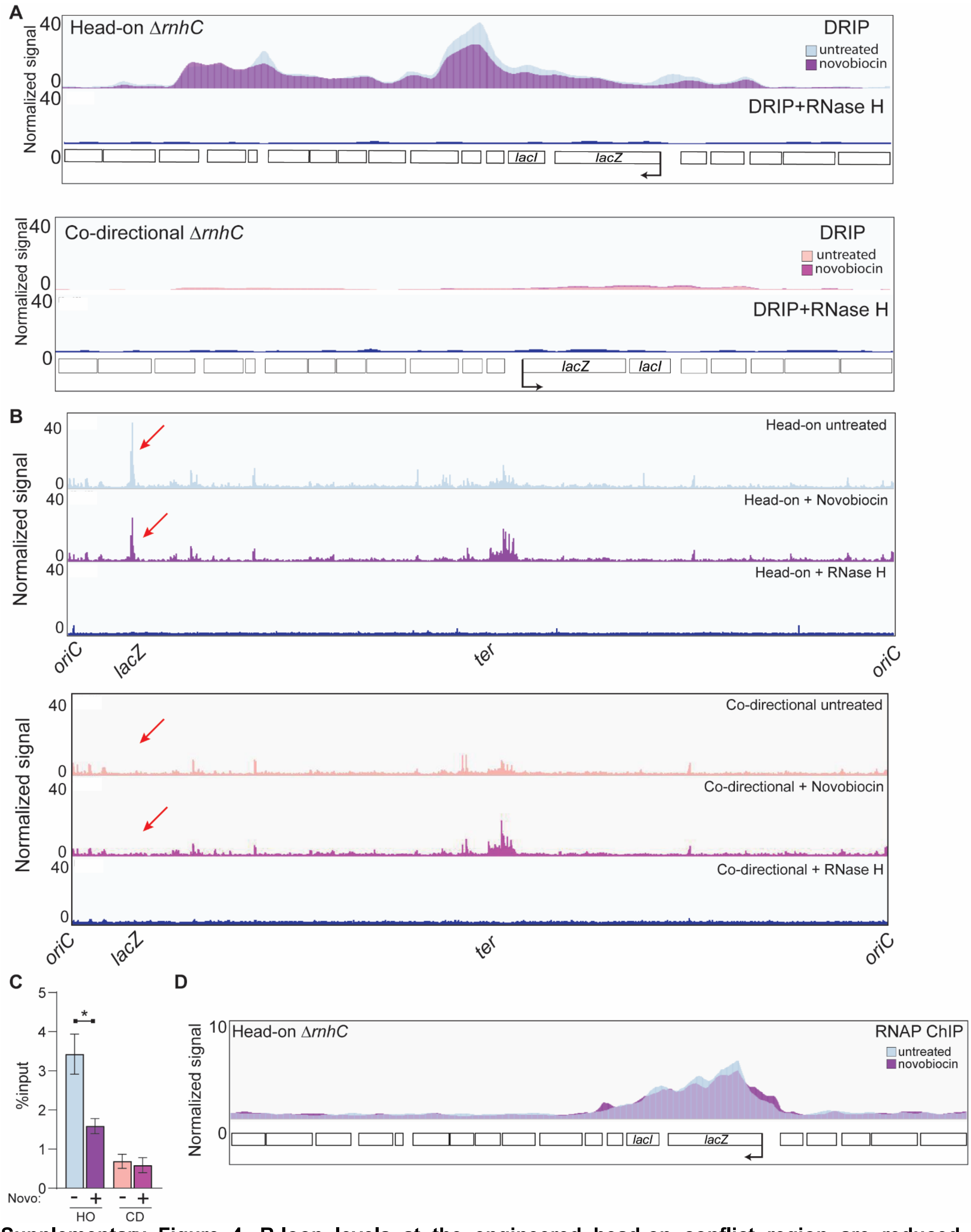

Supplementary Figure 4. R-loop levels at the engineered head-on conflict region are reduced in novobiocin treated cells. Related to Figure 4 (A) Replicate DRIP-Seq profiles of cells lacking RNase HIII harboring either a head-on (HO, blue, strain HM2043) or co-directional (CD, red, strain HM2044) *lacZ* engineered

conflict treated or untreated with novobiocin. Genome-wide maps are shown in **(B).** The bottom panel of all DRIP-Seq plots are the RNase H treated controls. **(C)** DRIP-qPCR analysis of the 3' region of *lacZ* with and without novobiocin treatment represented as percent input. The bars represent the mean and standard error of 4 biological replicates. (*) represents $p < 0.05$. **(D)** Representative RNAP ChIP-seq plot of Δ*rnhC* cells expressing the head-on engineered conflict untreated (blue) and treated with novobiocin (magenta). The conditions are shown as an overlay.

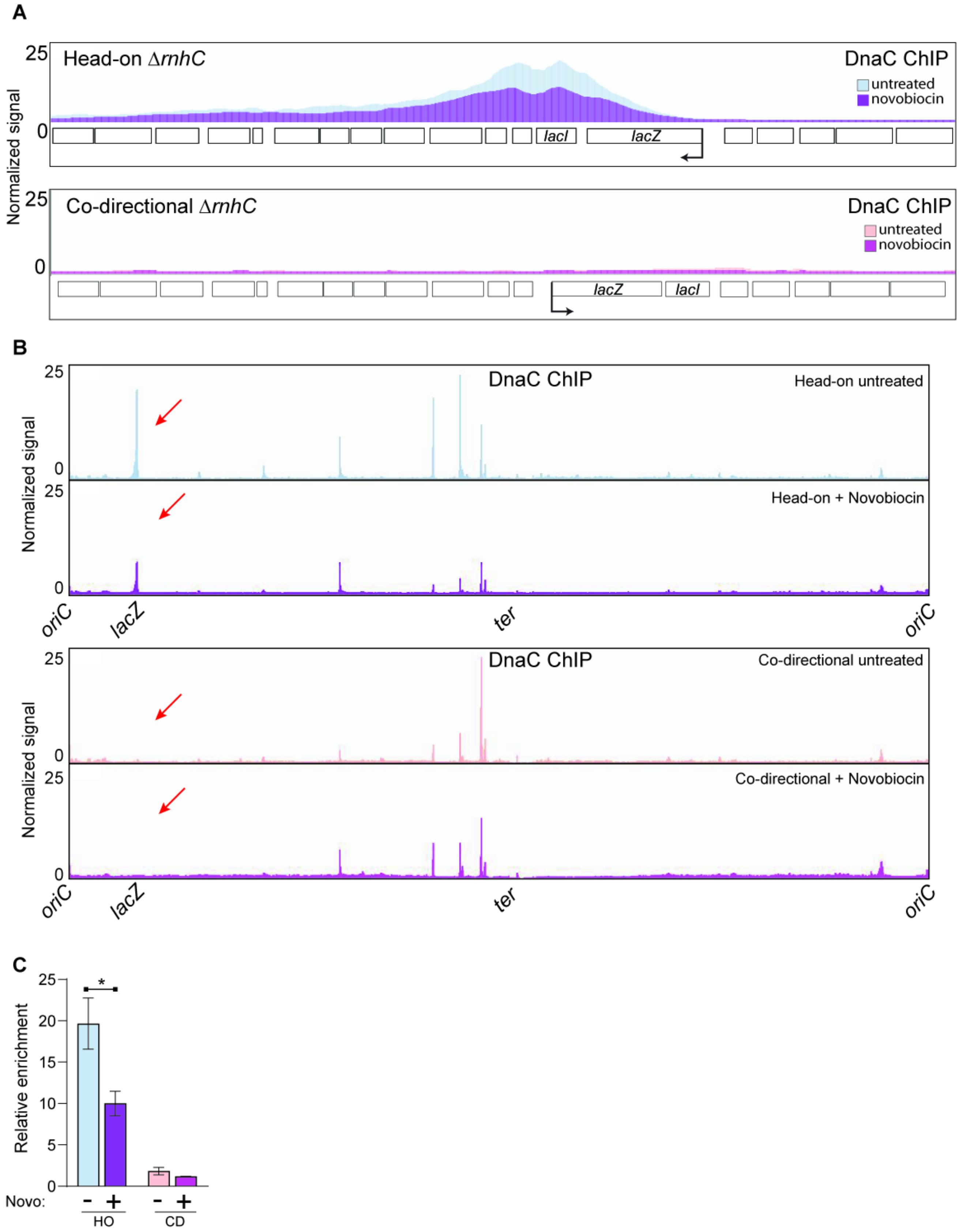

Supplementary Figure 5. Replication fork stalling due to R-loops at the engineered head-on conflict region is reduced with type II topoisomerase inhibition. Related to Figure 4. (A) Replicate DnaC ChIP-Seq profiles of cells lacking RNase HIII harboring either a head-on (HO, blue, strain HM2043) or co-directional (CD, red, strain HM2044) *lacZ* engineered conflict treated or untreated with novobiocin. Genome-wide maps are shown in **(B)**. **(C)** ChIP-qPCR analysis of the 3' region of *lacZ* with and without novobiocin treatment in cells lacking *rnhC*. The bars represent the mean and standard error of 4 biological replicates. (*) represents $p < 0.05$.

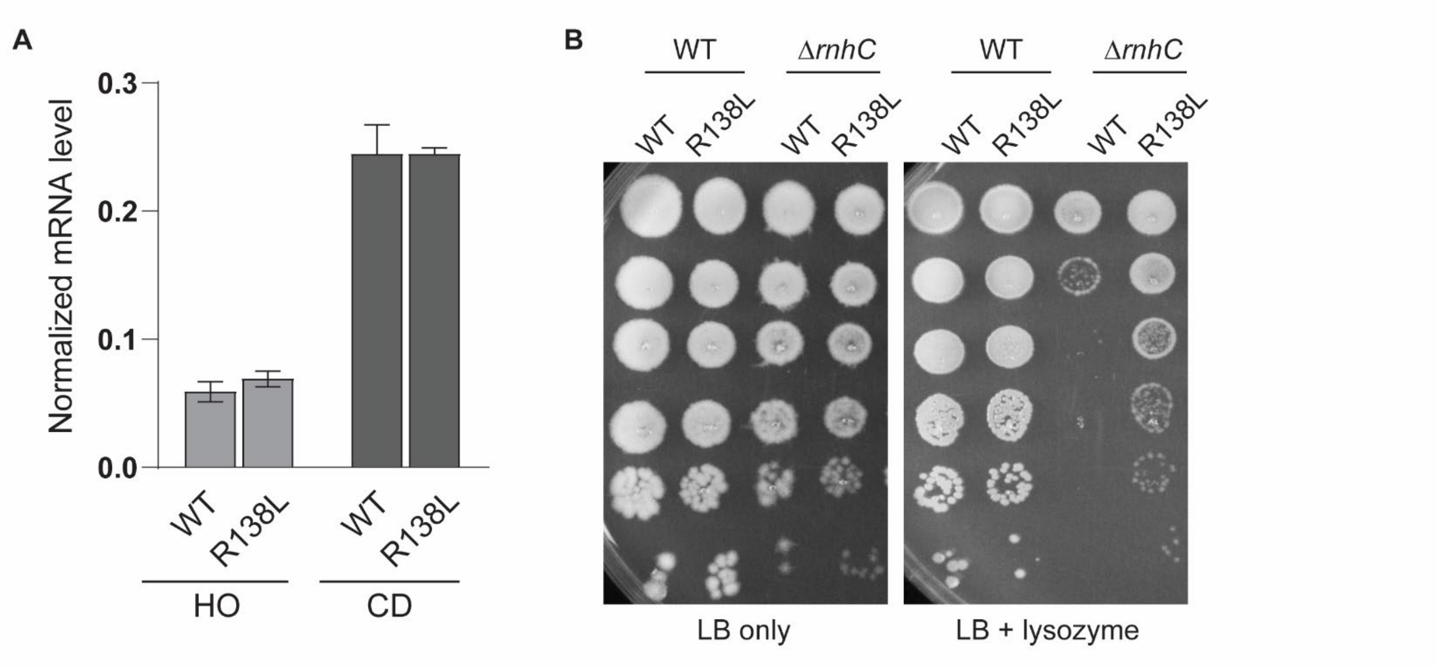

Supplementary Figure 6. Transcript levels of *lacZ* do not change in the R138L gyrase mutant, but stress response defects are rescued. Related to Figure 5. (A) qRT-PCR analysis measuring mRNA levels of *lacZ* relative to a housekeeping gene *rrs* in cells lacking RNase HIII with either the WT or R138L *gyrB* allele harboring either a head-on (HO, HM2043/HM4065) or co-directional (CD, HM2044/HM4066) *lacZ* engineered conflict. **(B)** Representative survival assays of WT cells or cells lacking RNase HIII with either the WT or R138L *gyrB* allele plated on either LB or LB containing 50 μg/ml of lysozyme.

Supplementary Table 1. Gyrase ChIP-Seq peaks identified genome-wide. Related to Figure 1.

start[a]	end[a]	peak score[b]	nearest gene
2197501	2199971	55457.4	yonT
1981403	1984331	42368.6	yoeD
327538	329328	33419.7	lacZ-lacI
3755421	3755631	3346.4	ywmC
1671341	1671441	720.7	sucD

a. Start and end genomic coordinates of peaks
b. Peak score calculated by Homer (IP compared to input)

Supplementary Table 2. ParC ChIP-Seq peaks identified genome-wide. Related to Figure 1.

start[a]	end[a]	peak score[b]	nearest gene
326665	331715	110502	*lacZ-lacI*
4189638	4190485	18922.1	*trmF*
5273	6189	17170.5	*gyrB*
4190470	4191199	15568.1	*trmE*
4187156	4187531	4796	*noc*
138158	138458	4278.9	*rpsS*
3935623	3936099	3978.1	*ilvK*
326461	326761	3445.6	*ycgB*
3936882	3937182	2945.5	*ilvK*
4191433	4191847	2442.2	*trmE*
4152542	4152992	1863.4	*yybL*
325129	325429	1773.5	*ycgA*
4153041	4153501	1677	*yybL*
4110512	4110890	1352.8	*yydD*
4186262	4186562	1217.4	*yyaB*
3646262	3646562	1010.7	*ggaB*
4153729	4154029	911.9	*yybK*

a. Start and end genomic coordinates of peaks
b. Peak score calculated by Homer (IP compared to input)

Supplementary Table 3. DnaC ChIP-Seq peaks identified genome-wide in wild-type cells. Related to Figure 2.

start[a]	end[a]	peak score[b]	nearest gene	average signal intensity (+/- std dev)[c]	
				untreated	treated
323023	331140	174468	*lacZ-lacI*	1.939(+/-0.073)	4.297(+/-1.11)
243	3049	41350.5	*dnaN*	3.3675(+/-0.415)	3.198(+/-1.075)
3936777	3937154	5900.9	*licH*	1.222(+/-0.094)	2.8335(+/-0.929)
1997029	1997908	4823.2	*yoxB*	15.537(+/-0.357)	6.4325(+/-2.001)
4193215	4193743	4189.2	*oxaAA*	2.387(+/-0.297)	2.518(+/-0.961)
3935747	3936047	2306.7	*ilvK*	1.394(+/-0.087)	2.495(+/-0.456)
1763564	1763864	1175.3	*cotE*	1.306(+/-0.058)	1.743(+/-0.673)

a. Start and end genomic coordinates of peaks
b. Peak score calculated by Homer (IP compared to input)
c. Average read density (ip/input) across replicate experiments within the peak regions.

Supplementary Table 4. Cut sites used for DRIP-Seq chromosome fragmentation at the engineered conflict locus. Related to Figure 4.

Fragment length	Enzyme sites
2076	EcoRI-5997-DraI-8073
1963	EcoRI-14898-EcoRV-16861
1481	DraI-19469-DraI-20950
1219	DraI-8753-EcoRV-9972
1178	EcoRV-16861-HindIII-18039
1074	HindIII-3686-DraI-4760
987	EcoRV-13788-DraI-14775
831	DraI-4760-DraI-5591
822	HindIII-2534-HindIII-3356
797	HindIII-353-HindIII-1150
779	HindIII-24016-HindIII-24795
675	DraI-18794-DraI-19469
663	DraI-8073-HindIII-8736
663	HindIII-11589-DraI-12252
657	DraI-23340-EcoRV-23997
624	DraI-22242-DraI-22866
599	DraI-13189-EcoRV-13788
551	EcoRV-21179-DraI-21730
512	DraI-21730-DraI-22242
490	DraI-12699-DraI-13189
474	DraI-22866-DraI-23340
451	DraI-2083-HindIII-2534
447	DraI-12252-DraI-12699
424	EcoRI-10979-HindIII-11403
395	HindIII-18039-EcoRI-18434
381	DraI-10598-EcoRI-10979
353	start-HindIII-353
307	EcoRV-9972-HindIII-10279
291	EcoRV-1792-DraI-2083
273	HindIII-3413-HindIII-3686
263	DraI-18531-DraI-18794
237	HindIII-1150-DraI-1387
229	DraI-20950-EcoRV-21179
211	HindIII-10387-DraI-10598
208	EcoRV-25043-end
186	HindIII-11403-HindIII-11589
177	EcoRV-1615-EcoRV-1792
175	HindIII-24868-EcoRV-25043
162	DraI-1387-EcoRV-1549
155	DraI-5591-DraI-5746
126	DraI-5746-DraI-5872
125	DraI-5872-EcoRI-5997
123	DraI-14775-EcoRI-14898
108	HindIII-10279-HindIII-10387
97	EcoRI-18434-DraI-18531
73	HindIII-24795-HindIII-24868

66	EcoRV-1549-EcoRV-1615
57	HindIII-3356-HindIII-3413
19	EcoRV-23997-HindIII-24016
17	HindIII-8736-DraI-8753

Supplementary Table 5. DRIP-Seq peaks identified genome-wide in Δ*rnhC* cells. Related to Figure 4.

start[a]	end[a]	peak score[b]	nearest gene	average signal intensity (+/- std dev)[c]	
				untreated	treated
326514	328288	104923.5	*lacZ-lacI*	31.8915(+/-3.2885)	23.727(+/-2.849)
320595	321068	10352.6	*yceK*	21.464(+/-2.932)	11.642(+/-2.876)
2195721	2196840	9727.1	*yonX*	17.494(+/-5.43)	13.774(+/-3.09)
2205720	2206116	4312.5	*yonK*	6.863(+/-0.655)	12.199(+/-1.829)
2199715	2200167	3476	*yonR*	15.2025(+/-5.6295)	9.519(+/-2.333)
3156556	3156856	3125.6	*thiT*	4.044(+/-0.814)	4.1605(+/-1.4335)
2195342	2195728	2761.9	*yopA*	15.323(+/-5.207)	14.4445(+/-3.7375)
2203688	2203988	2586.7	*yonN*	5.282(+/-0.317)	8.936(+/-0.976)
942449	942749	2149.6	*spo0M*	11.3975(+/-0.0865)	7.1765(+/-0.7225)
1894389	1894764	2115.7	*cotC*	8.957(+/-1.865)	3.2955(+/-0.4705)
2199497	2199797	1706.9	*yonS*	14.1335(+/-5.7965)	10.0885(+/-2.0395)
2225847	2226332	1652.3	*youB*	8.043(+/-1.796)	8.5895(+/-2.7065)
2200908	2201208	1612.7	*yonO*	11.68(+/-3.27)	8.126(+/-1.701)
2165020	2165320	1053.2	*yorF*	4.725(+/-0.678)	3.674(+/-0.432)
2184825	2185125	1011.7	*yopQ*	6.2645(+/-1.7985)	4.491(+/-1.051)
100138	100438	906.2	*ctsR*	1.714(+/-0.265)	1.7595(+/-0.4875)

a. Start and end genomic coordinates of peaks
b. Peak score calculated by Homer (IP compared to input)
c. Average read density (ip/input) across replicate experiments within the peak regions.

Supplementary Table 6. DnaC ChIP-Seq peaks identified genome-wide in Δ*rnhC* cells. Related to Figure 4.

start[a]	end[a]	peak score[b]	nearest gene	average signal intensity (+/- std dev)[c]	
				untreated	treated
318699	331480	325183.6	*lacZ-lacI*	12.169(+/-2.378)	7.8815(+/-1.3485)
1892711	1896089	50375	*cotC*	16.607(+/-4.462)	4.157(+/-0.433)
1995150	1998713	49744.2	*rtp*	11.6215(+/-1.5545)	6.8645(+/-0.5535)
1762517	1765736	46372.4	*cotE*	12.973(+/-2.762)	3.7645(+/-1.4635)
1311141	1312741	23008.7	*xkdC*	9.7685(+/-4.1445)	8.5015(+/-1.2095)
317781	318312	10485.9	*niaP*	4.736(+/-1.022)	3.743(+/-0.756)
317248	317513	4803.1	*niaP*	4.5265(+/-1.3085)	3.2575(+/-0.5705)
316756	316980	2770	*yceH*	4.2335(+/-1.4415)	2.786(+/-0.585)
2014811	2015077	1986.8	*yoaM*	4.3525(+/-0.6565)	3.5695(+/-0.5045)
2015730	2016078	1863.4	*yozS*	5.3065(+/-0.4825)	3.3(+/-1.395)
942138	942288	983.8	*spo0M*	4.2355(+/-1.2235)	1.812(+/-0.072)
1892228	1892378	513.1	*thyA*	3.5535(+/-0.1195)	1.0505(+/-0.2205)
941681	941831	499.6	*spo0M*	3.579(+/-0.523)	1.777(+/-0.256)
1896289	1896439	486.1	*ynzB*	3.802(+/-0.718)	1.5965(+/-0.1455)

a. Start and end genomic coordinates of peaks
b. Peak score calculated by Homer (IP compared to input)
c. Average read density (ip/input) across replicate experiments within the peak regions.
