# Supplementary material for: Topological stress is responsible for the detrimental outcomes of head-on replication-transcription conflicts
Source: Cell Rep. Author manuscript; Available in PMC 2021 Mar 23. (PMC7986047; doi:10.1016/j.celrep.2021.108797)
Supplement: 2 [file NIHMS1679665-supplement-2.pdf]

Cell Reports

Article

Topological stress is responsible for the detrimental outcomes of head-on replication-transcription conflicts

Graphical Abstract

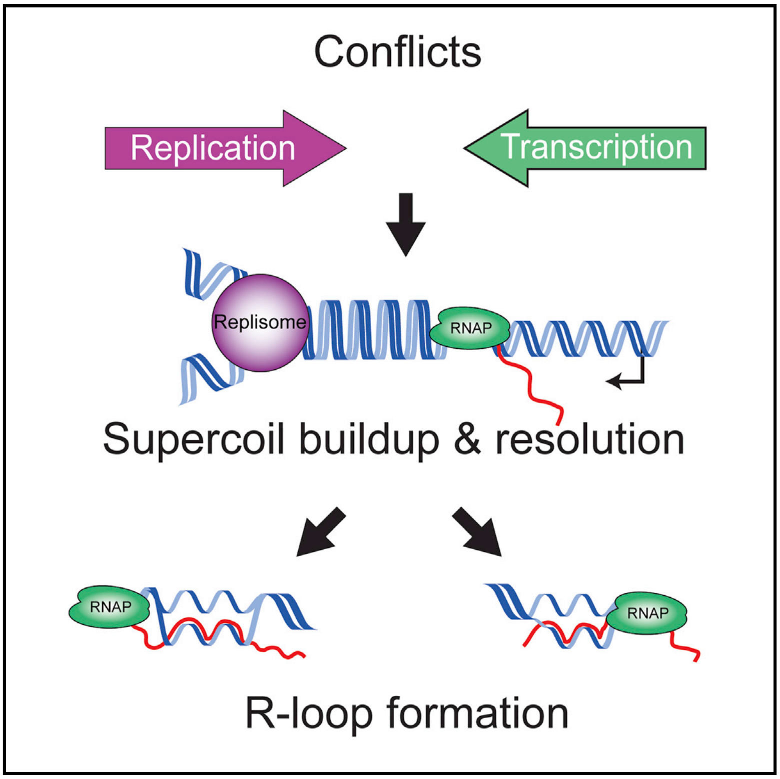

Highlights

- Type II topoisomerases act at head-on replication-transcription conflict regions
- Cells with severe head-on conflicts are sensitive to the inhibition of topoisomerases
- Type II topoisomerase inhibition increases replisome stalling at head-on genes
- Supercoil resolution at head-on conflict regions drives R-loop formation

Authors

Kevin S. Lang, Houra Merrikh

Correspondence

houra.merrikh@vanderbilt.edu

In brief

Lang and Merrikh show that resolution of head-on, but not codirectional, conflicts between replication and transcription machineries requires type II topoisomerases, suggesting that a fundamental difference between the two types of conflicts is supercoil buildup in DNA. Furthermore, they show that supercoil resolution at head-on conflict regions drives R-loop formation.

Lang & Merrikh, 2021, Cell Reports *34*, 108797
March 2, 2021 © 2021 The Author(s).
https://doi.org/10.1016/j.celrep.2021.108797

CellPress

Cell Reports

CellPress
OPEN ACCESS

Article

Topological stress is responsible for the detrimental outcomes of head-on replication-transcription conflicts

Kevin S. Lang[1] and Houra Merrikh[1,2,*]
[1]Department of Biochemistry, Light Hall, Vanderbilt University, Nashville, TN, USA
[2]Lead contact
*Correspondence: houra.merrikh@vanderbilt.edu
https://doi.org/10.1016/j.celrep.2021.108797

SUMMARY

Conflicts between the replication and transcription machineries have profound effects on chromosome duplication, genome organization, and evolution across species. Head-on conflicts (lagging-strand genes) are significantly more detrimental than codirectional conflicts (leading-strand genes). The fundamental reason for this difference is unknown. Here, we report that topological stress significantly contributes to this difference. We find that head-on, but not codirectional, conflict resolution requires the relaxation of positive supercoils by the type II topoisomerases DNA gyrase and Topo IV, at least in the Gram-positive model bacterium *Bacillus subtilis*. Interestingly, our data suggest that after positive supercoil resolution, gyrase introduces excessive negative supercoils at head-on conflict regions, driving pervasive R-loop formation. Altogether, our results reveal a fundamental mechanistic difference between the two types of encounters, addressing a long-standing question in the field of replication-transcription conflicts.

INTRODUCTION

Transcription and DNA replication occur simultaneously on the same template. The lack of spatiotemporal separation between these two processes leads to conflicts between them multiple times every replication cycle. The replication and transcription machineries can encounter each other either head-on or codirectionally. Codirectional conflicts occur when genes are transcribed on the leading strand whereas head-on conflicts occur when genes are transcribed on the lagging strand. It has been demonstrated that head-on conflicts are more deleterious than codirectional conflicts in that they cause increased mutagenesis, DNA breaks, replisome stalling, and replication restart across diverse organisms (Chappidi et al., 2020; Dutta et al., 2011; French, 1992; Hamperl et al., 2017; Lang et al., 2017; Merrikh and Merrikh, 2018; Merrikh et al., 2011; Million-Weaver et al., 2015a, 2015b; Mirkin and Mirkin, 2005; Paul et al., 2013; Pomerantz and O'Donnell, 2010; Prado and Aguilera, 2005; Wang et al., 2007). Despite many insightful studies into these inevitable encounters, the fundamental question regarding why head-on conflicts are more detrimental than codirectional conflicts remains unanswered. It is perplexing that encounters between the same two machineries (the replication machinery or the replisome and RNA polymerase [RNAP]) can have such different outcomes simply due to orientation.

Topological constraints could explain why head-on conflicts are more deleterious than codirectional conflicts. Unwinding of DNA during transcription generates positively supercoiled DNA ahead and negatively supercoiled DNA behind RNAP (Liu and Wang, 1987; Wu et al., 1988). Similarly, during replication, positive supercoils accumulate in front of the replisome (Hiasa and Marians, 1996; Postow et al., 1999; Vos et al., 2011). The resolution of this supercoiled DNA is critical for both transcription and replication to proceed efficiently (Khodursky et al., 2000). In a codirectional conflict, the positive supercoiling generated in front of the replisome would encounter the negative supercoiling produced from active RNAPs ahead. This would most likely cause a net neutral change in local supercoiling levels. However, during a head-on conflict, the positive supercoiling generated ahead of the replisome would encounter the positive supercoiling produced by RNAP. Therefore, in a head-on conflict, there may be a transient buildup of positive supercoils that has the potential to change the fundamental mechanics of the replisome and RNAP. Such changes could stall the replisome, leading to disassembly and changing the dynamics of RNAP movement and associated mRNAs. These predictions suggest that torsional stress could be the key driver of conflict severity, and therefore, this model must be tested.

Another important question is whether topoisomerases are critical conflict resolution factors. The resolution of supercoils in all organisms requires topoisomerases (Champoux, 2001; Vos et al., 2011; Wang, 2002). In bacteria, there are two topoisomerases that relax positive supercoils, DNA gyrase and Topo IV. DNA gyrase and Topo IV are both required for replication fork progression *in vivo* (Ashley et al., 2017; Crisona et al., 2000; Khodursky et al., 2000; Peng and Marians, 1993; Vos et al., 2011). Topo IV also plays a critical role in the resolution of catenanes (intertwined chromosomes) as well as the separation of sister

This is an open access article under the CC BY license (http://creativecommons.org/licenses/by/4.0/).

chromatids during segregation (Hiasa and Marians, 1996; Zechiedrich and Cozzarelli, 1995). If the torsional stress hypothesis is correct, then type II topoisomerases should be critical conflict resolution factors, yet this question has not been addressed.

Here, we report that type II topoisomerases preferentially associate with head-on genes and that cells harboring engineered head-on conflicts are sensitized to type II topoisomerase inhibitors. Accordingly, we find that conditional depletion of either gyrase or Topo IV is deleterious to cells experiencing engineered head-on conflicts. Inhibition of type II topoisomerase activity leads to increased stalling of the replisome when it approaches a gene transcribed in the head-on, but not codirectional, orientation. Remarkably, however, our data strongly suggest that negative supercoil introduction by DNA gyrase at head-on conflict regions is responsible for the formation of R-loops in these regions. Consistent with this finding, we observe that in cells lacking the RNase HIII enzyme, which resolves R-loops, inhibition of type II topoisomerases lowers R-loop abundance and alleviates R-loop-induced replisome stalling at head-on genes. Furthermore, an allele of gyrase that is strongly defective in the introduction of negative supercoils completely rescues the head-on-conflict-induced lethality of cells lacking RNase HIII. This rescue is also observed when cells are exposed to lysozyme-induced cell wall stress, which is well known to induce a number of endogenous genes, including head-on operons that range from 3 to 6 kb in length (Guariglia-Oropeza and Helmann, 2011).

RESULTS

Type II topoisomerases preferentially associate with a head-on, but not codirectional, engineered conflict region

The relaxation of both positive and negative supercoils is an essential process in all cells. In *Bacillus subtilis*, relaxation of positive supercoils is accomplished by the activity of either gyrase or Topo IV (Ashley et al., 2017; Crisona et al., 2000; Postow et al., 2001a; Vos et al., 2011). If the model of positive supercoil accumulation at head-on conflict regions is correct, then these enzymes should preferentially associate with a head-on conflict region. To test this hypothesis, we measured gyrase and Topo IV enrichment genome-wide using chromatin immunoprecipitation followed by deep sequencing (ChIP-seq). In order to study the effects of topology at head-on conflict regions, we took advantage of several different tightly controlled engineered conflict systems, all of which were integrated onto the chromosome. In each of these systems, the same exact gene (e.g., *lacZ*) was inserted onto the same locus in either the head-on or codirectional orientation with respect to replication. To control for gene expression levels, both the head-on and codirectional versions of the gene were placed under the control of the same promoter. In particular, we chose promoters (e.g., $P_{spank(hy)}$) that achieve transcrition levels that are close to those of essential and highly transcribed genes that are oriented codirectionally (see Figure S6 for quantification of levels relative to rRNA for the codirectional gene). Such high levels of transcription for the majority of head-on genes are only achieved under specific conditions, such as during exposure to environmental stresses (Guariglia-Oropeza and Helmann, 2011; Lang et al., 2017; Mostertz et al., 2004; Nicolas et al., 2012). Therefore, we did not expect to see enrichment of type II topoisomerases at endogenous head-on genes during growth in rich media. Lastly, our previous data indicated that transcription levels are the same in both orientations in this engineered conflict system (Lang et al., 2017).

In order to measure the relative association of type II topoisomerases with the conflict regions, we used a GFP fusion to the GyrA subunit of gyrase (Tadesse and Graumann, 2006) and constructed a 3xMyc fusion to the ParC subunit of Topo IV. We expressed an IPTG-inducible *lacZ* gene in either the head-on or codirectional orientation and performed ChIP-seq experiments to obtain a high-resolution map of the association of type II topoisomerases with both the genome and, specifically, the engineered conflict regions.

We found that both gyrase and Topo IV are preferentially enriched at the engineered conflict locus when the orientation of *lacZ* is head on (Figures 1A, 1B, S1A, S1C, and S1E). Compared to other peaks identified across the genome, the gyrase and Topo IV enrichment at the engineered head-on conflict was one of the largest peaks, suggesting a significant topological problem in the engineered conflict locus (Figures S1B and S1C; Tables S1 and S2). Importantly, this enrichment was transcription dependent. When we measured enrichment of these topoisomerases using ChIP-qPCR, we found that in the absence of the inducer, IPTG, the levels of topoisomerases at the engineered conflict regions were similar in the two orientations (Figures 1C and 1D). Furthermore, we confirmed that the GyrA signal was specific by performing control ChIPs of GFP only (unfused to GyrA) and found no enrichment at the *lacZ* gene in either orientation (Figure 1E). It is noteworthy that we utilized standard formaldehyde crosslinking for the GyrA ChIPs. However, we were unable to ChIP ParC using formaldehyde. The ParC association was only detectable when we performed the ChIP assays using ciprofloxacin crosslinking, which specifically crosslinks active type II topoisomerases on DNA.

Inhibition of type II topoisomerases increases the association of DnaC (the replicative helicase) at head-on, but not codirectional, genes

In *E. coli*, gyrase and Topo IV promote replication fork progression (Khodursky et al., 2000). If torsional stress is a major problem at head-on conflict regions, then subtle inhibition of these topoisomerases should lead to increased replication fork stalling at these loci. We tested this hypothesis by performing ChIP-seq of the replicative helicase, DnaC, as a proxy for replication stalling. If fork progression is unimpeded, then the distribution of DnaC enrichment should be equal along the genome in asynchronous bacterial cultures. We have demonstrated previously that DnaC enrichment is a good proxy for replication fork stalling (Lang et al., 2017; Merrikh et al., 2015, 2011). To inhibit type II topoisomerase activity, we used subinhibitory doses of the antibiotic novobiocin. Novobiocin is a competitive inhibitor of type II topoisomerase ATPase activity (Hardy and Cozzarelli, 2003; Maxwell, 1993; Sugino et al., 1978). We performed ChIP-seq experiments in which we measured the association of DnaC genome-wide, which includes the engineered conflict loci in media with and without sublethal concentrations of novobiocin

CellPress
OPEN ACCESS

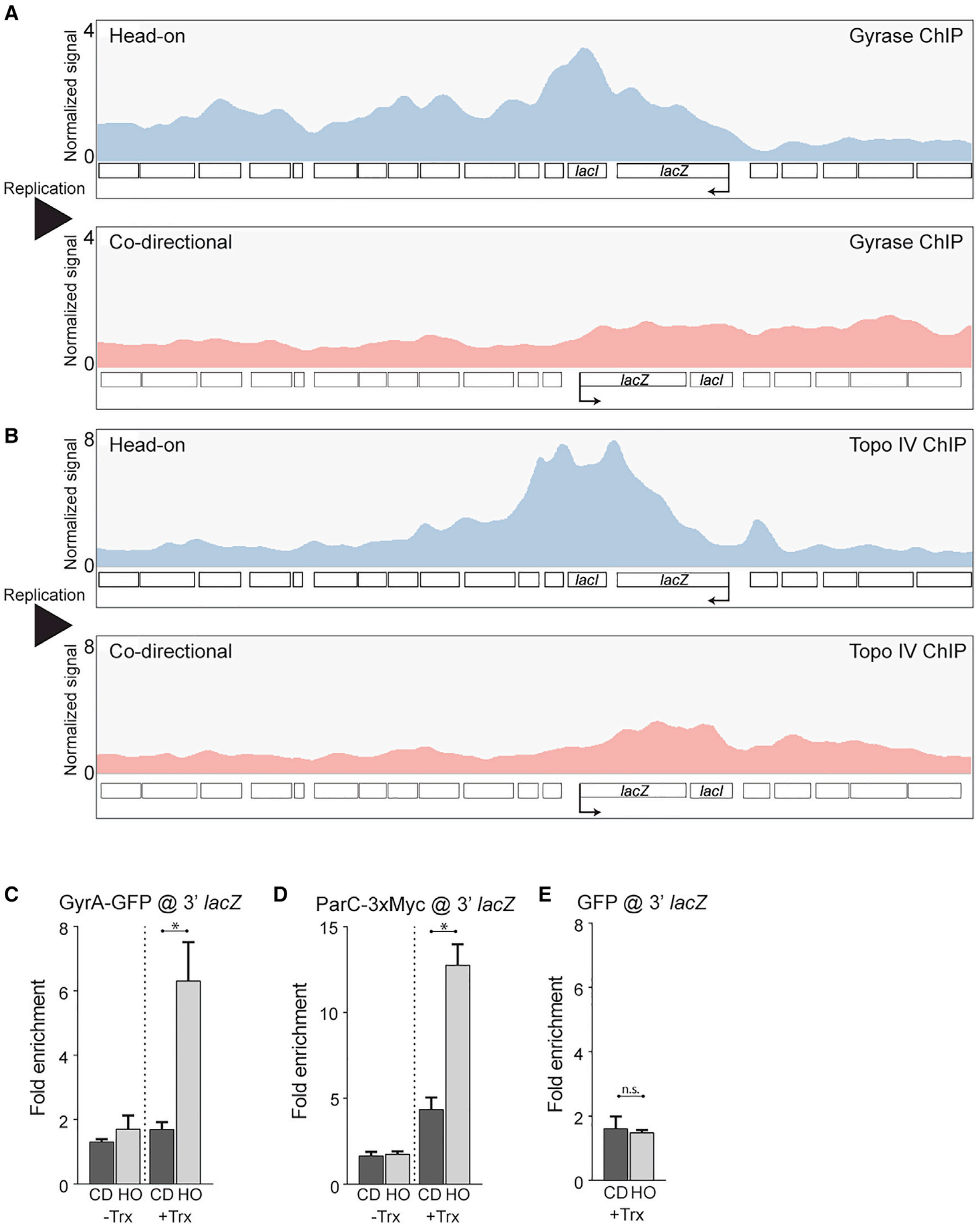

Figure 1. Type II topoisomerases are enriched at head-on genes

(A and B) DNA gyrase (A) and Topo IV (B) ChIP-seq profiles of cells carrying either a head-on (HO; blue, strain HM3863 [gyrase], HM4074 [ParC]) or codirectional (CD; red, strain HM3864 [gyrase], HM4075 [ParC]) *lacZ* engineered conflict. Normalized signal is the read depth of immunoprecipitate (IP)/input normalized to the total number of reads. The direction of DNA replication is left to right. Direction of transcription is indicated by the promoter arrow on *lacZ*.

(C–E) ChIP-qPCR analysis of DNA gyrase (C), Topo IV (D), and GFP (E) in cells carrying either an HO (strain HM3863 [gyrase], HM4074 [ParC], HM3019 [GFP]) or CD (strain HM3864 [gyrase], HM4075 [ParC], HM3020 [GFP]) *lacZ* engineered conflict. "Trx" refers to transcription of the engineered conflict. Relative enrichment is the signal of *lacZ* normalized to input relative to a control locus, *yhaX*, normalized to input. Bars represent the mean and standard error. *$p < 0.05$; n.s., not significant.

See also Figure S1 and Tables S1 and S2.

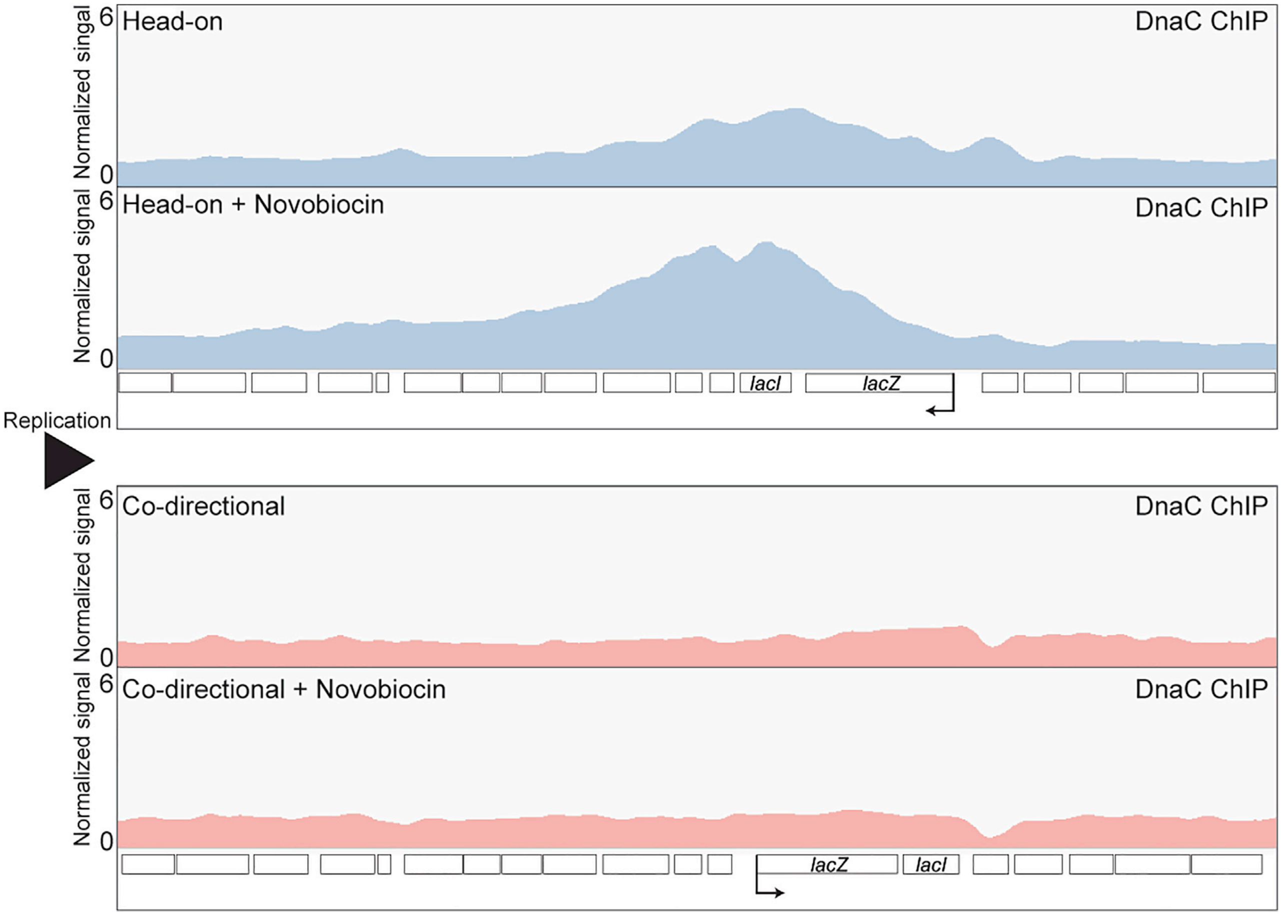

Figure 2. Type II topoisomerase inhibition results in increased DnaC accumulation at HO genes

DnaC ChIP-seq profiles of cells carrying either an HO (blue, strain HM3863) or CD (red, strain HM2864) *lacZ* engineered conflict, with and without novobiocin treatment (375 ng/mL). Normalized signal is the read depth of IP/input normalized to the total number of reads. The direction of DNA replication is left to right. Direction of transcription is indicated by the promoter arrow on *lacZ*.

See also Figure S2 and Table S3.

(375 ng/mL). Genome-wide, there are very few DnaC peaks, and those we identified happen to be near the terminus, as previously shown (Tables S2 and S6; Figure S2) (Merrikh et al., 2015; Smits et al., 2011). We found that when the cells were treated with novobiocin, there was an increase in DnaC enrichment at the head-on, but not codirectional, conflict region (Figures 2 and S2; Table S3). These results suggest that without type II topoisomerase activity, topological problems at head-on genes can impede replication.

Inhibition of type II topoisomerase activity compromises cell survival specifically in the presence of a strong head-on conflict

We previously showed that in the absence of critical conflict resolution factors, head-on conflicts can significantly compromise survival efficiency (Lang et al., 2017; Merrikh et al., 2015; Million-Weaver et al., 2015b). If type II topoisomerases are indeed important for conflict resolution, then the inhibition of these enzymes should impact survival of cells experiencing head-on conflicts. To test this hypothesis, we measured survival efficiency using colony-forming units (CFUs) of cells containing the engineered conflicts in the head-on or codirectional orientation upon chronic treatment with various concentrations of novobiocin. In the absence of novobiocin, there was no difference in survival efficiency of cells containing the engineered conflict in either orientation and regardless of whether the *lacZ* gene was transcribed (Figure 3A). When the cells were plated on novobiocin, again, there was no difference in survival efficiency between cells carrying the head-on or codirectional *lacZ* when transcription was off. However, when transcription was turned on, the cells carrying the head-on-, but not codirectionally, oriented *lacZ* gene were sensitized to low doses of novobiocin. The effects of head-on conflicts on survival, in response to inhibition of type II topoisomerases, was not specific to the chromosomal location or the nature of the gene used to induce the conflict.

In order to control for potential indirect effects of genomic context, chromosomal location, and sequence, we performed similar survival experiments using a second engineered conflict system. In this system, we inserted a different transcription unit, the *luxABCDE* operon, onto the opposite (right) arm of the

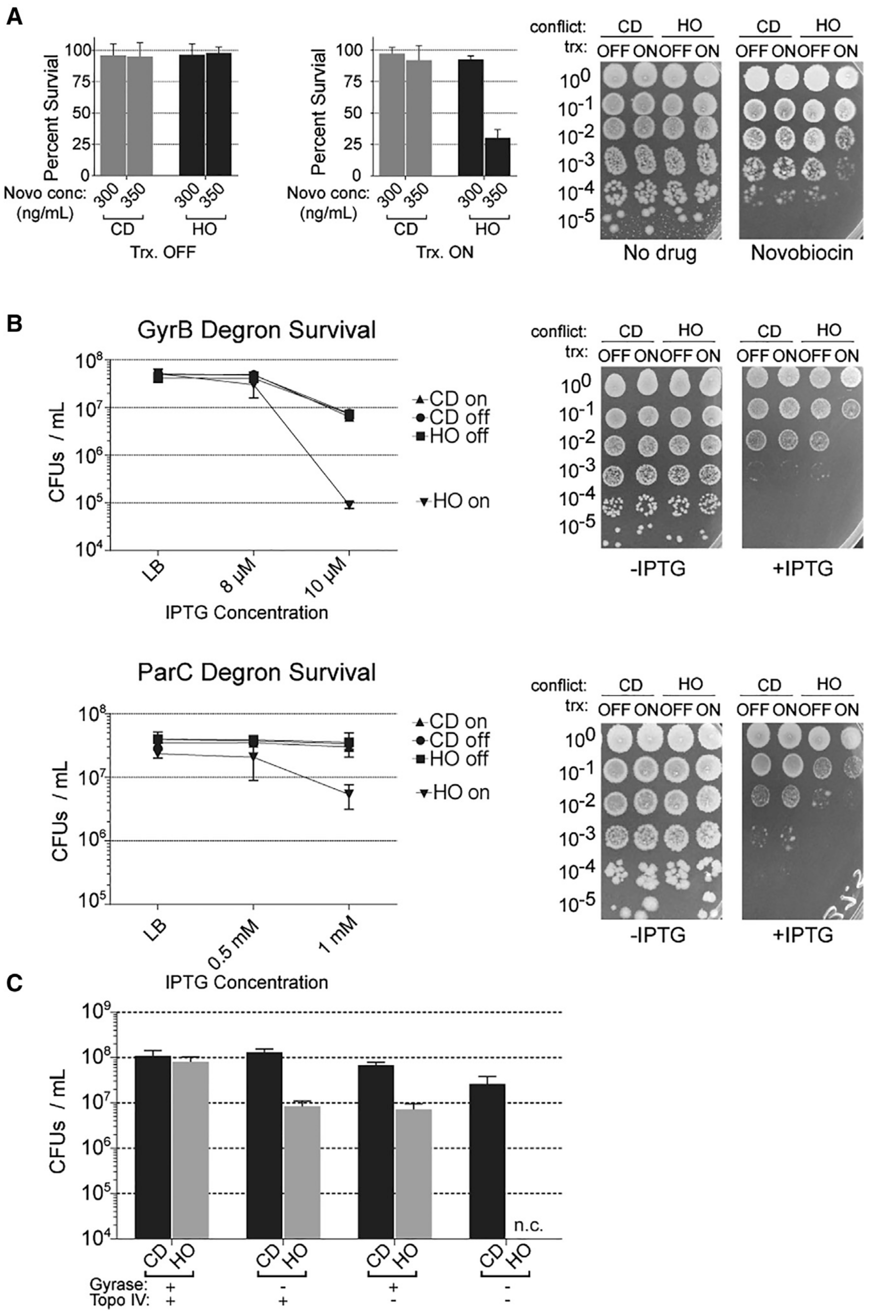

Figure 3. DNA gyrase and Topo IV act in parallel to resolve HO conflicts

(A) Survival of cells harboring either a repressed (HO, HM640; CD, HM1794) or constitutively transcribed (HO, HM211; CD, HM1795) *lacZ* engineered conflict plated on LB or LB supplemented with novobiocin (300 or 350 ng/mL). Bar graphs are quantification (mean and standard deviation) of three independent biological replicates.

(B) Survival after conditional (IPTG-dependent) depletion of either gyrase or Topo IV in cells harboring either a repressed (HO, HM1951/HM1467; CD, HM1949/HM1468) or constitutively transcribed (HO, HM1952/HM1450; CD, HM1950/HM1469) *lacZ* engineered conflict plated on LB or LB supplemented with IPTG (as indicated; plates shown are representative plates of the highest concentration).

(C) Survival of cells harboring a novobiocin-resistant *gyrB* allele, a conditional gyrase depletion (IPTG-dependent) system and a constitutively transcribed (HO, HM2420; CD, HM2421) *lacZ* engineered conflict plated on LB or LB supplemented with novobiocin (7 μg/mL), LB supplemented with IPTG (10 μM), or both novobiocin and IPTG.

"Trx" refers to transcription of the engineered conflict. n.c., no countable colonies.

See also Figure S3.

chromosome. We performed the survival experiments with this system as described above. The results of these experiments were consistent with the *lacZ* system; there was a survival defect in cells containing the *luxABCDE* operon, but only when this transcription unit was in the head-on orientation and only when the genes were transcribed (Figure S3).

Both gyrase and Topo IV are critical for the resolution of head-on conflicts

Novobiocin has activity against both gyrase and Topo IV, although the affinity of the drug for Topo IV is much weaker than that for gyrase (Peng and Marians, 1993; Sugino et al., 1978). It was unclear from our survival assays whether the survival defects were a result of inhibition of only gyrase or Topo IV or both. It is likely that only gyrase activity is inhibited at the concentrations of novobiocin we used in our experiments. However, it cannot be ruled out that Topo IV activity is also inhibited to some extent under these conditions. To directly determine the contribution of each of the two enzymes to conflict resolution, we adapted a conditional degradation system (Griffith and Grossman, 2008) to specifically deplete the GyrB subunit of gyrase or the ParC subunit of Topo IV. This system is induced by IPTG, and we confirmed the depletion by western blot (Figure S3C). In order to detect potentially subtle differences in survival of our engineered conflict strains, we used concentrations of IPTG that only slightly depleted GyrB and ParC and subtly impacted survival of wild-type cells (gyrase and Topo IV are essential, so a complete depletion cannot be used here). We then tested the survival of cells carrying engineered conflicts under these conditions, but now the engineered conflicts expressed *lacZ* from a different promoter, P_{xis}, which is constitutively active. The "transcription off" control for this engineered conflict is achieved through the use of a strain in which this promoter is constitutively off. In

both the GyrB and ParC degron systems, we found that without IPTG, there was no difference in survival efficiency (although colonies become smaller, likely due to depleting essential enzymes) in any of the engineered conflict strains. When we specifically depleted GyrB in cells carrying the codirectional conflict, transcription of *lacZ* made no difference in survival efficiency. In cells carrying the head-on engineered conflict, however, there were significant defects in survival only when the transcription of the engineered conflict was on (Figure 3B). Similarly, when we depleted ParC, there was an ~90% reduction in the number of CFUs when comparing strains with transcriptionally active versus inactive head-on *lacZ* (Figure 3B).

In order to address whether gyrase and Topo IV act together or in parallel, we constructed a strain that had a mutation in the *gyrB* gene that conferred a high level of resistance to novobiocin (R138L). In this background, novobiocin treatment can only impact Topo IV. In this same strain, we fused the *gyrB* gene to the *ssrA* tag in order to deplete gyrase with our degron system. We found that low concentrations of IPTG (GyrB depletion) or high levels of novobiocin (Topo IV inhibition) both led to a survival defect in the strain carrying the head-on, but not codirectional, conflict (Figure 3C). When we treated cells with both IPTG and novobiocin, the cells expressing the head-on *lacZ* gene were not viable (Figure 3C). This result indicates that gyrase and Topo IV are the only two factors that can resolve the torsional stress problem at head-on conflict regions.

Inhibition of type II topoisomerases reduces R-loop formation at head-on conflict regions

There is evidence in the literature that topoisomerase activity can influence R-loop formation, at least *in vitro* and in human cells (Massé and Drolet, 1999; Tuduri et al., 2009). Furthermore, our results described above strongly suggest that DNA topology is a serious problem at head-on conflict regions. Given our prior results that R-loops contribute to many of the detrimental outcomes of head-on conflicts, we decided to investigate whether resolution of head-on conflicts by topoisomerases influence R-loop formation. We tested this hypothesis by directly measuring R-loop levels at the conflict regions in strains lacking RNase HIII (Lang et al., 2017; Ohtani et al., 1999; Randall et al., 2018). We performed DNA-RNA hybrid immunoprecipitations coupled to deep sequencing (DRIP-seq) experiments using the S9.6 antibody, which recognizes RNA:DNA hybrids. We treated our samples in parallel with RNase H in order to ensure specificity for RNA:DNA hybrids and calculated the percent yield in our pull-downs (Figures 4A and S4). Consistent with what we have measured previously using qPCR (Lang et al., 2017), we found more R-loops when the *lacZ* gene was expressed in the head-on orientation compared to the codirectional orientation (Figures 4A and S4). The DRIP peak at the engineered head-on conflict is the largest peak relative to the others found genome-wide (Figure S4; Table S5). Using a conservative enrichment of 20-fold over the input, we found 16 other prominent peaks around the genome, many of which were near or spanning the most highly transcribed genes (Table S5).

We then used DRIP-seq to measure R-loops in cells treated with low levels of novobiocin to subtly reduce the activities of both type II topoisomerases. Remarkably, we found that when type II topoisomerases are inhibited, R-loop levels are reduced at the head-on conflict region (Figures 4A and S4; Table S5). The lack of a difference in RpoB occupancy at the engineered conflict regions with this amount of novobiocin treatment indicated that the lowered R-loop levels are not simply due to reduced expression of the head-on *lacZ* gene (Figure S4D). We found that the RpoB signal was much more constrained to the head-on conflict locus compared to the R-loop signal in that region. One interpretation of this unexpected result is that diffusion of supercoils leads to R-loop formation away from the immediate vicinity of the conflict.

Inhibiting type II topoisomerases reduces replisome stalling at the engineered head-on conflict in cells that cannot process R-loops

R-loops at head-on genes stall the replisome in many different organisms (Hamperl et al., 2017; Lang et al., 2017; Prado and Aguilera, 2005). If type II topoisomerase activity is driving R-loop formation at head-on genes, then treating cells with low doses of novobiocin should reduce replisome stalling at head-on conflict regions in cells lacking RNase HIII. We tested this hypothesis using DnaC ChIP-seq, as described above. As we published previously, we found that there is a preferential association of DnaC with head-on versus codirectional conflict regions, and this difference is significantly increased in cells lacking RNase HIII (Figure 4B). This DnaC ChIP signal at head-on conflict regions, in cells lacking RNase HIII, corresponds to complete replication fork stalling at that locus (Lang et al., 2017). When we treated cells with low amounts of novobiocin to inhibit topoisomerase activity, there was a decrease in DnaC enrichment at the head-on conflict region (Figures 4B and S5; Table S6). This result suggests that the type II topoisomerases are responsible for R-loop-mediated replisome stalling at head-on conflict regions.

Inhibiting type II topoisomerases rescues lethality of an engineered head-on conflict in the absence of RNase HIII

We previously showed that increased stalling due to unresolved R-loops at head-on genes is lethal (Lang et al., 2017). If topoisomerase activity is driving R-loop formation at head-on genes, then limiting that activity should increase the viability of cells that contain an engineered head-on conflict and lack RNase HIII. We tested this model by measuring the viability of cells lacking RNase HIII and expressing either the head-on or codirectional *lacZ* in the presence of low concentrations of novobiocin. As expected, cells with the codirectional engineered conflict had no growth defect when the *lacZ* gene was induced with IPTG. In contrast, cells expressing the *lacZ* gene in the head-on orientation had significant cell survival defects. Remarkably, chronic novobiocin exposure rescued these defects in a dose-dependent manner (Figure 4C). Altogether, these results suggest that the resolution of head-on conflicts by type II topoisomerase activity is driving R-loop formation, which affects cell viability.

Introduction of negative supercoils by gyrase promotes R-loop formation at head-on conflict regions

Novobiocin inhibits both gyrase and Topo IV activity. However, gyrase is much more sensitive to novobiocin than Topo IV

CellPress
OPEN ACCESS

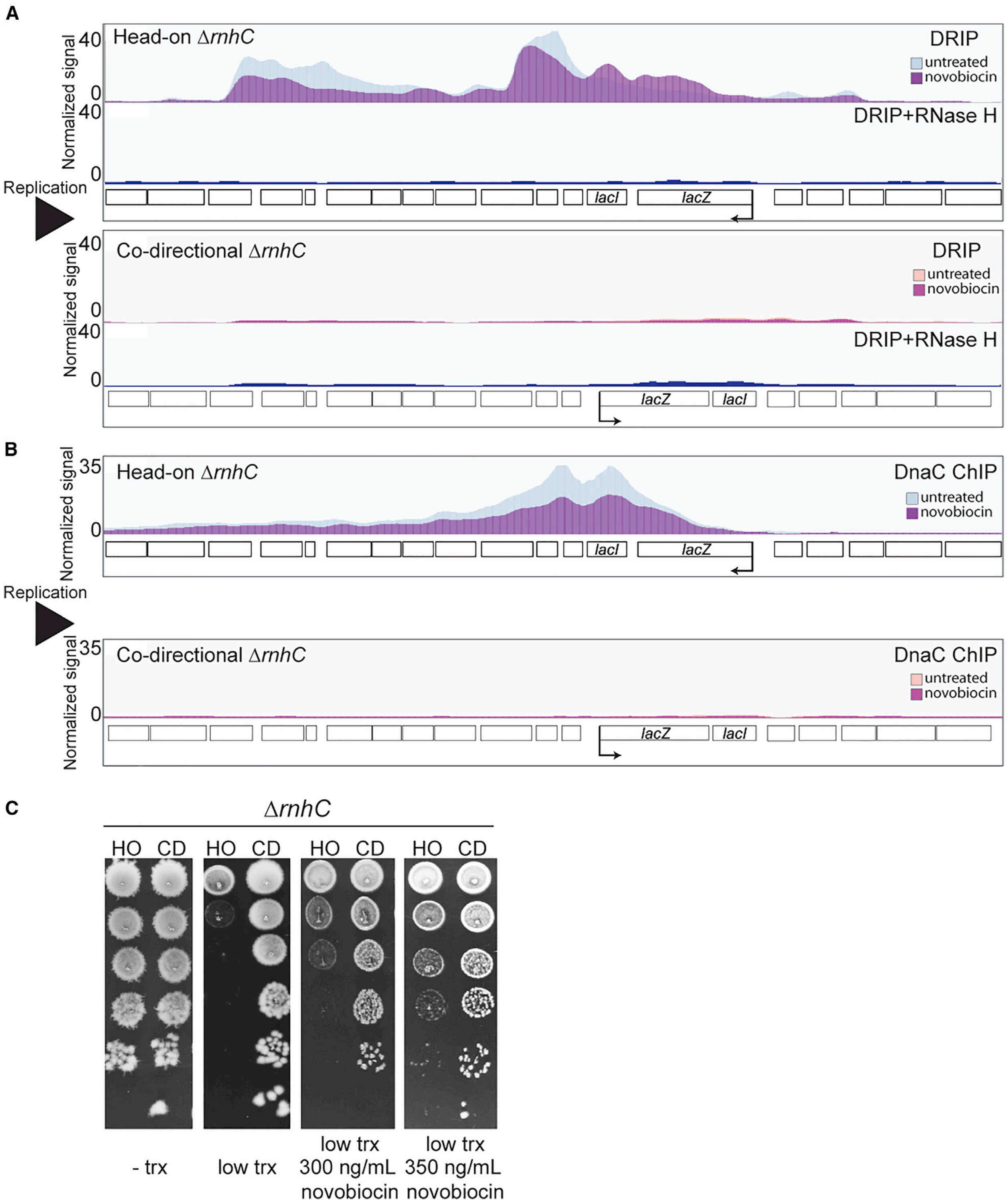

Figure 4. Resolution of HO conflicts by Type II topoisomerases promotes the formation of R-loops

(A and B) DRIP-seq (A) and DnaC (B) ChIP-seq profiles of cells lacking RNase HIII harboring either an HO (blue, strain HM2043) or CD (strain HM2044) *lacZ* engineered conflict treated or untreated with novobiocin. The bottom panel of both DRIP-seq profiles is the RNase-H-treated control. Normalized signal is the read depth of IP/input normalized to the total number of reads.

(C) Survival of cells lacking RNase HIII harboring either an HO (strain HM2043) or CD (strain HM2044) *lacZ* engineered conflict treated or untreated with novobiocin. See also Figures S4 and S5 and Tables S4–S6.

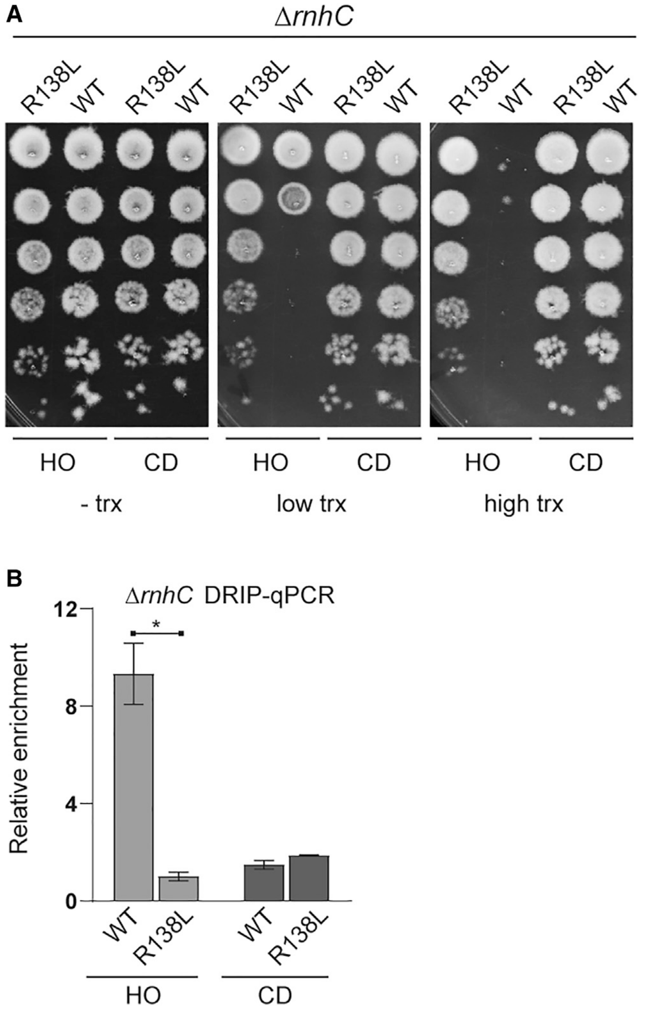

Figure 5. DNA gyrase impacts R-loop formation at HO genes

(A) Survival of cells lacking RNase HIII with either the wild-type (WT) or R138L *gyrB* allele harboring either an HO (HM2043/HM4065) or CD (HM2044/HM4066) *lacZ* engineered conflict.

(B) DRIP-qPCR analysis of cells lacking RNase HIII with either the WT or R138L *gyrB* allele harboring either a HO (HM2043/HM4065) or CD (HM2044/HM4066) *lacZ* engineered conflict. Relative enrichment is the signal of *lacZ* normalized to input relative to a control locus *yhaX* normalized to input. Bars represent the mean and standard error of four biological replicates. *$p < 0.05$.

See also Figure S6.

(Khodursky et al., 2000; Sugino et al., 1978; Levine et al., 1998). We wondered whether the decreased R-loop levels was due to inhibition of gyrase and not inhibition of Topo IV or pleiotropic effects of novobiocin. Gyrase has two activities: (1) relaxation of positive supercoiling and (2) introduction of negative supercoiling (Vos et al., 2011). Both *in vitro* and *in vivo*, R-loops have been shown to form more readily (or are more stable) in the presence of gyrase (Drolet et al., 1994, 1995; Massé and Drolet, 1999). This is likely due to the introduction of negative supercoiling by gyrase, as negatively supercoiled DNA will energetically favor R-loop formation, although recent work has suggested that highly positively increased supercoiling could also impact R-loop formation (Stolz et al., 2019). We tested this model by utilizing the *gyrB* (R138L) mutant, which has reduced ATPase activity and thus has a 10-fold reduction in the ability to introduce negative supercoils (Contreras and Maxwell, 1992; Gross et al., 2003). Whether and/or how much this mutation impacts the positive supercoil relaxation activity of gyrase has not been assessed. However, Topo IV can resolve torsional stress at conflict regions in parallel to gyrase, as we showed above. Therefore, even if the positive supercoil relaxation activity of gyrase is impacted by the R138L mutation, the major effect of this mutation at the conflict region will be a loss of negative supercoil introduction. We used survival assays to measure viability of Δ*rnhC* strains containing the mutant *gyrB* in the presence of either the head-on- or codirectionally oriented conflicts. As expected, there was no effect of transcription on the viability of the cells carrying the codirectional engineered conflict. Consistent with our previous work, we found that induction of the engineered conflict was completely lethal when it was oriented head-on to replication. Remarkably, we found that the *gyrB* R138L mutation completely rescued this lethality (Figure 5A). This rescue was not due to altered transcript levels due to the R138L mutation (Figure S6A). We tested whether this rescue was due to the reduction of R-loops at the conflict region by measuring R-loop association levels directly by DRIP-qPCRs. In cells lacking RNase HIII, consistent with what we have previously reported, we measured roughly 5-fold higher R-loop signal at the head-on compared to the codirectional *lacZ* (Figure 5B). When we measured R-loops in cells with the R138L *gyrB* mutation, the R-loop levels were similar at the head-on and codirectional conflict regions. These results demonstrate that it is specifically the introduction of negative supercoils by gyrase at head-on conflict regions that leads to the formation (and/or stability) of R-loops.

When we chronically expose cells to various stresses, including cell wall stress induced by lysozyme exposure, we observe a defect in survival of Δ*rnhC* cells relative to wild-type (Lang et al., 2017). We previously proposed that this phenotype is a result of conflict-induced problems at head-on stress response genes. Here, we tested whether the supercoiling induced R-loop formation is a potential problem at endogenous head-on genes, leading to the observed phenotypic defects. We find that the R138L *gyrB* mutation suppresses stress response defects of the Δ*rnhC* strain. We hypothesize that this phenotypic rescue is due to a decrease in R-loop formation at lysozyme resistance genes that are encoded head on (Figure S6B) (see Guariglia-Oropeza and Helmann, 2011 for more information regarding key lysozyme resistance genes). Any gene that responds to lysozyme stress in the codirectional orientation should not experience excess R-loop formation. This is true for all stressors; although stress response genes are encoded in both orientations, only the head-on-oriented ones will experience excess R-loop formation upon induction. Altogether, these results are consistent with the idea that our engineered conflict systems are representative of what occurs at endogenous head-on genes when they are induced. Future experiments should be performed to further investigate the impact of conflicts at endogenous genes, although delineating the impact of various stresses, gene length, and transcription levels will require significant effort.

CellPress
OPEN ACCESS

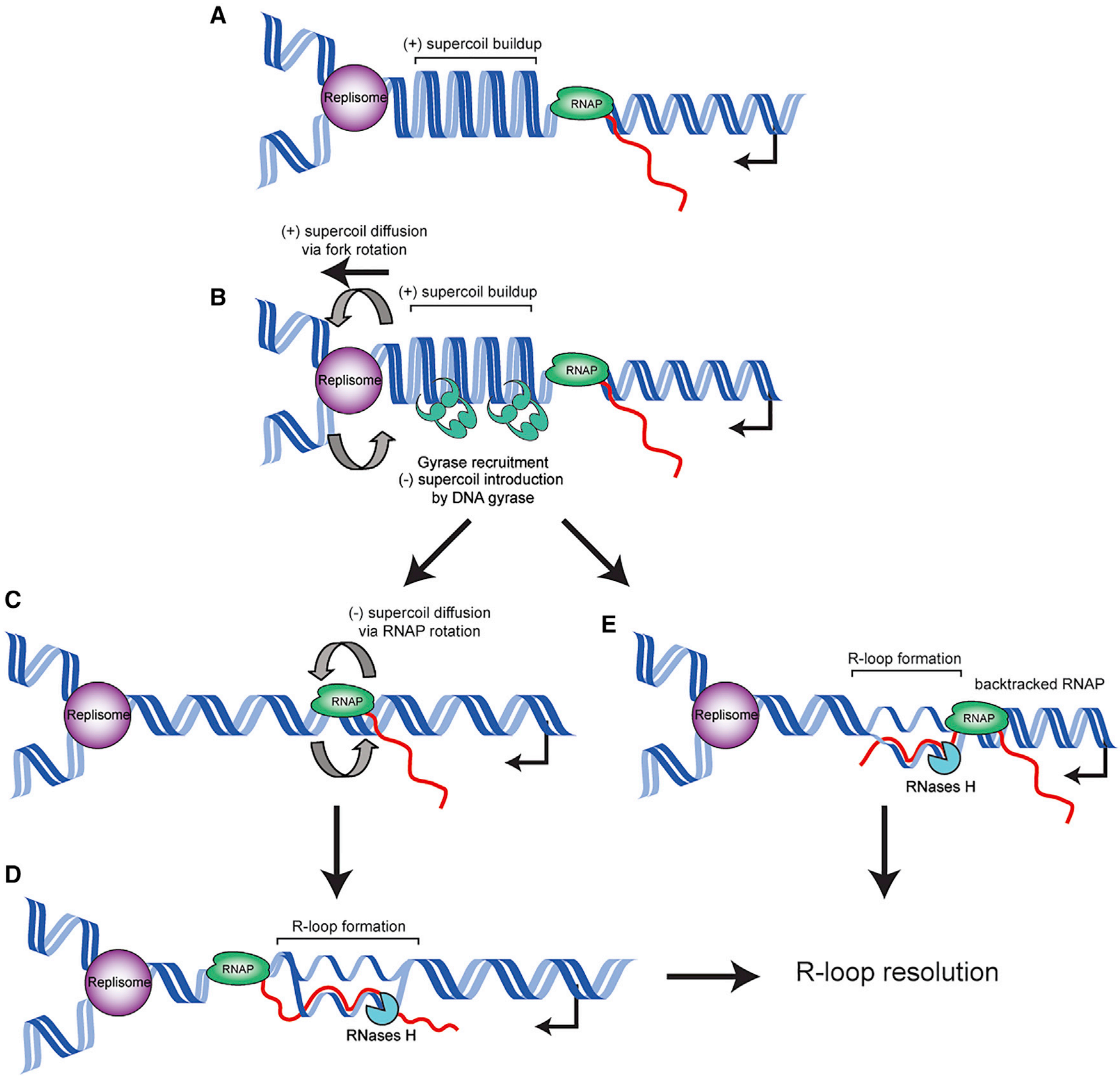

Figure 6. Proposed model for topological changes and R-loop formation at HO conflict regions

(A) As the replisome and HO transcription unit converge, positive supercoils accumulate in between the two machineries.

(B) DNA gyrase resolves the positive supercoil buildup. The replisome also likely spins to relieve the torsional strain, producing catenanes behind the replication fork, which are resolved by Topo IV.

(C) Gyrase activity rapidly converts the conflict region to negatively supercoiled DNA, causing RNAP to spin about its axis. Negative supercoils diffuse behind RNAP.

(D) The diffused negative supercoils drive R-loop formation behind RNAP, which are resolved by RNase H enzymes.

(E) Alternatively, topological problems cause RNAP to backtrack, allowing an R-loop to form from the exposed 3′ end of the nascent mRNA.

DISCUSSION

The problem of replication-transcription conflicts exists in all domains of life. Gene-orientation-dependent effects of transcription on DNA replication have been a topic of interest since the analysis of genome organization in *E. coli* (Brewer, 1988), followed by the discovery that strong head-on transcription slows replication significantly more than codirectional transcription (French, 1992). However, why the orientation of transcription relative to DNA replication matters has remained a mystery. The protein makeup of the two machineries is the same in both orientations, yet the direction in which they encounter each other has profound downstream effects. In this work, we address at least one of the major underlying reasons for this difference.

Our results strongly suggest that positive supercoils build up at head-on conflict regions. We also find that gyrase activity at head-on genes drives R-loop formation. These results can be explained by several potential models. First, a "spin diffusion" model could explain our observations. In this model, excess negative supercoils generated by gyrase promote R-loop formation through the diffusion of the supercoils past RNAPs (Figure 6). This process would be initially triggered by positive supercoil

buildup between the replication and transcription machineries at head-on conflict regions, which is rapidly removed by type II topoisomerases. Gyrase would then lead to the generation of hypernegatively supercoiled DNA (Ashley et al., 2017; Drlica, 1992; Drolet et al., 1994, 1995; Lynch and Wang, 1993). This increase in negative supercoiling would then diffuse through RNAP spinning about its axis (Nudler, 2009, 2012; Ma et al., 2013; Lodge et al., 1989). Alternatively, given that gyrase is recruited over a broad area, it could introduce negative supercoils across the region without a need for RNAP spinning. It has previously been shown that supercoils are constrained when RNAP is unable to spin due to expression of membrane-bound protein-coding gene (Lodge et al., 1989). In a second model, the sudden release of torsional strain by type II topoisomerases could cause RNAP to rapidly progress, generating excessive negative supercoils and R-loop formation (Kuzminov, 2018). Additionally, R-loops could form in front of RNAP due to RNAP backtracking, exposing the 3′ end of the nascent mRNA (Nudler, 2012). This exposed 3′ end could reanneal to the coding DNA strand, forming an R-loop. These models are not mutually exclusive and could all be contributing to R-loop formation and stability at head-on gene regions.

In our previous work, we observed that cells can no longer replicate the chromosome after encountering R-loops at our engineered head-on conflict systems (Lang et al., 2017). One possible explanation for this lethality is that the R-loops completely block the replisome, either because the replicative helicase cannot unwind them or because they stabilize RNAP such that it cannot be removed, resulting in a barrier to the replication fork. Alternatively, many stalled forks in the same genomic region could lead to toxic recombination events and/or production of unresolvable replication intermediates that can be lethal to cells (Magner et al., 2007). Whatever the root cause, the viability of cells with a highly transcribed gene in the head-on orientation requires resolution of R-loops.

The importance of Topo IV in resolving head-on conflicts adds a second dimension to our findings. The observations that Topo IV is important for conflict resolution can be interpreted in two ways: (1) Topo IV helps relax positive supercoils at conflict regions, and/or (2) the increased torsional stress leads to the formation of catenanes by inducing replisome spinning about its axis (Bermejo et al., 2007; Keszthelyi et al., 2016; Schalbetter et al., 2015). Given that there is a significant amount of literature showing that Topo IV is critical for catenane resolution, we favor the second possibility (Espeli and Marians, 2004; Levine et al., 1998; Zechiedrich and Cozzarelli, 1995). These models, however, are not mutually exclusive.

In addition to the topology model, one hypothesis that could explain gene orientation effects of conflicts is the strand specificity of where the replicative helicase resides (lagging strand in bacteria and leading strand in eukaryotes) (Gómez-González and Aguilera, 2019; Hamperl and Cimprich, 2016). This model could explain why the two different types of conflicts have differential consequences. However, the discovery that R-loops are a major problem in head-on, but not codirectional, conflicts in both bacteria and mammalian cells undermines this model (Hamperl et al., 2017; Lang et al., 2017). The replicative helicase moves on the lagging strand in bacteria whereas it moves on the leading strand in mammalian cells, yet the fundamental problem of R-loop enrichment in head-on conflicts remains the same across these species. Therefore, gene-orientation-specific problems are unlikely to stem from this particular architectural feature of the replisome complex. On the other hand, production of positive supercoils by the replication and transcription machineries is a universal feature and therefore could be the fundamental mechanism underlying gene-orientation-specific effects of replication-transcription conflicts. Recent work in human cells suggested that topology plays a role in R-loop formation at head-on gene regions, demonstrating the conservation of the results presented here (Promonet et al., 2020).

It is clear from this work, as well as that of others, that after some encounters with the transcription machinery, replication stalls, the replisome collapses, and replication progression requires restart proteins (Mangiameli et al., 2017; Merrikh et al., 2011). However, the extent to which the fork is remodeled and whether there is replication fork reversal after a head-on conflict are not yet clear. Previous studies have implied that in head-on conflicts, the replication fork reverses and is subsequently processed by recombination proteins (Chappidi et al., 2020; De Septenville et al., 2012; Million-Weaver et al., 2015b). Furthermore, it has been shown *in vitro* that replication forks reverse in response to positive supercoil accumulation (Postow et al., 2001b). Given that at least in eukaryotic systems supercoiling can push the fork back, our data are consistent with the model that conflicts lead to replication fork reversal due to positive supercoil buildup.

We previously proposed that the head-on orientation is retained for some genes as a mechanism to increase mutagenesis and promote gene-specific evolution (Merrikh and Merrikh, 2018; Paul et al., 2013). Further work showed that the increased mutagenesis of head-on genes is driven by R-loops in wild-type cells (Lang et al., 2017). Given that gyrase activity is facilitating R-loop formation, our results suggest that the activity of this enzyme, albeit indirectly, leads to increased mutagenesis. Interestingly, as our group and others have shown, the full capacity of gyrase to introduce negative supercoils is not essential for viability (Gross et al., 2003). Why then is this function conserved? We speculate that the introduction of negative supercoils by gyrase is evolutionarily beneficial. In particular, we previously showed that head-on genes, including many of the critical stress response genes (which are functionally enriched in the head-on orientation), evolve faster than codirectional genes. Under selection, these head-on genes will be highly transcribed, gaining beneficial mutations faster than if they were codirectionally oriented, simply due to a conflict-induced increase in mutation rates. If those beneficial mutations are obtained through negative supercoil introduction by gyrase (and downstream R-loop formation), then this property of gyrase would be retained over evolutionary time despite the fact that it is not immediately necessary for viability. In other words, the activity of gyrase to introduce negative supercoils would hitchhike along in cells that have rapidly adapted to their environment by obtaining beneficial mutations relatively quickly through this mechanism.

In this work, we discovered (what appears to be) the main source of gene-orientation-specific problems in replication-transcription conflicts. We also unraveled an intriguing feature of

topoisomerases that, in the big picture, could place them into a category of evolutionarily beneficial factors that increase mutagenesis. These findings highlight the fundamental importance and influence of conflicts and DNA supercoiling on cellular physiology, genome organization, and adaptation.

STAR★METHODS

Detailed methods are provided in the online version of this paper and include the following:

- KEY RESOURCES TABLE
- RESOURCE AVAILABILITY
  - Lead contact
  - Materials availability
  - Data and code availability
- EXPERIMENTAL MODEL AND SUBJECT DETAILS
  - Bacterial strains and growth conditions
  - Plasmid and strain constructions
- METHOD DETAILS
  - Viability assays
  - Slot blot analysis
  - Chromatin immunoprecipitation assays (ChIPs)
  - DNA:RNA hybrid immunoprecipitation assays (DRIPs)
  - RNA extraction and cDNA preparation
  - Next generation sequence analysis
- QUANTIFICATION AND STATISTICAL ANALYSIS

SUPPLEMENTAL INFORMATION

Supplemental Information can be found online at https://doi.org/10.1016/j.celrep.2021.108797.

ACKNOWLEDGMENTS

We would like to thank Christopher Merrikh and the other members (past and present) of the Merrikh lab, as well as Neil Osheroff and Felipe Cortés Ledesma for helpful discussions. The authors would also like to thank Sara Mangiameli for revising the manuscript and Peter Graumann for sharing strains. This work was supported by National Institutes of Health awards DP2GM110773 and R01GM128191-02 to H.M., Research Royalty Funds from the University of Washington to H.M., the 5T32-AI055396-13 Bacterial Pathogenesis Training Grant (University of Washington) award to K.S.L., and the 1F32 AI140557-01 Ruth L. Kirschstein National Research Postdoctoral award to K.S.L. We would like to dedicate this paper to the late James Champoux, who discovered Top1 and discussed this project with us on various occasions during our time at the University of Washington.

AUTHOR CONTRIBUTIONS

K.S.L. and H.M. designed and performed the experiments, analyzed the data, and wrote the paper.

DECLARATION OF INTERESTS

The authors declare no competing interests.

Received: August 1, 2019
Revised: December 14, 2020
Accepted: February 5, 2021
Published: March 2, 2021

REFERENCES

Ashley, R.E., Dittmore, A., McPherson, S.A., Turnbough, C.L., Jr., Neuman, K.C., and Osheroff, N. (2017). Activities of gyrase and topoisomerase IV on positively supercoiled DNA. Nucleic Acids Res. *45*, 9611–9624.

Bermejo, R., Doksani, Y., Capra, T., Katou, Y.-M., Tanaka, H., Shirahige, K., and Foiani, M. (2007). Top1- and Top2-mediated topological transitions at replication forks ensure fork progression and stability and prevent DNA damage checkpoint activation. Genes Dev. *21*, 1921–1936.

Bolger, A.M., Lohse, M., and Usadel, B. (2014). Trimmomatic: a flexible trimmer for Illumina sequence data. Bioinformatics *30*, 2114–2120.

Brehm, S.P., Staal, S.P., and Hoch, J.A. (1973). Phenotypes of pleiotropic-negative sporulation mutants of Bacillus subtilis. J Bacteriol *115*, 1063–1070, PMID: 4199504; PMCID: PMC246354. https://doi.org/10.1128/JB.115.3.1063-1070.1973.

Brewer, B.J. (1988). When polymerases collide: replication and the transcriptional organization of the E. coli chromosome. Cell *53*, 679–686.

Champoux, J.J. (2001). DNA topoisomerases: structure, function, and mechanism. Annu. Rev. Biochem. *70*, 369–413.

Chappidi, N., Nascakova, Z., Boleslavska, B., Zellweger, R., Isik, E., Andrs, M., Menon, S., Dobrovolna, J., Balbo Pogliano, C., Matos, J., et al. (2020). Fork Cleavage-Religation Cycle and Active Transcription Mediate Replication Restart after Fork Stalling at Co-transcriptional R-Loops. Mol. Cell *77*, 528–541.e8.

Contreras, A., and Maxwell, A. (1992). gyrB mutations which confer coumarin resistance also affect DNA supercoiling and ATP hydrolysis by Escherichia coli DNA gyrase. Mol. Microbiol. *6*, 1617–1624.

Crisona, N.J., Strick, T.R., Bensimon, D., Croquette, V., and Cozzarelli, N.R. (2000). Preferential relaxation of positively supercoiled DNA by E. coli topoisomerase IV in single-molecule and ensemble measurements. Genes Dev. *14*, 2881–2892.

De Septenville, A.L., Duigou, S., Boubakri, H., and Michel, B. (2012). Replication fork reversal after replication-transcription collision. PLoS Genet. *8*, e1002622.

Drlica, K. (1992). Control of bacterial DNA supercoiling. Mol. Microbiol. *6*, 425–433.

Drolet, M., Bi, X., and Liu, L.F. (1994). Hypernegative supercoiling of the DNA template during transcription elongation in vitro. J. Biol. Chem. *269*, 2068–2074.

Drolet, M., Phoenix, P., Menzel, R., Massé, E., Liu, L.F., and Crouch, R.J. (1995). Overexpression of RNase H partially complements the growth defect of an Escherichia coli delta topA mutant: R-loop formation is a major problem in the absence of DNA topoisomerase I. Proc. Natl. Acad. Sci. USA *92*, 3526–3530.

Dutta, D., Shatalin, K., Epshtein, V., Gottesman, M.E., and Nudler, E. (2011). Linking RNA polymerase backtracking to genome instability in E. coli. Cell *146*, 533–543.

Espeli, O., and Marians, K.J. (2004). Untangling intracellular DNA topology. Mol. Microbiol. *52*, 925–931.

French, S. (1992). Consequences of replication fork movement through transcription units in vivo. Science *258*, 1362–1365.

García-Rubio, M., Aguilera, P., Lafuente-Barquero, J., Ruiz, J.F., Simon, M.-N., Geli, V., Rondón, A.G., and Aguilera, A. (2018). Yra1-bound RNA-DNA hybrids cause orientation-independent transcription-replication collisions and telomere instability. Genes Dev. *32*, 965–977.

Gómez-González, B., and Aguilera, A. (2019). Transcription-mediated replication hindrance: a major driver of genome instability. Genes Dev. *33*, 1008–1026.

Griffith, K.L., and Grossman, A.D. (2008). Inducible protein degradation in Bacillus subtilis using heterologous peptide tags and adaptor proteins to target substrates to the protease ClpXP. Mol. Microbiol. *70*, 1012–1025.

Gross, C.H., Parsons, J.D., Grossman, T.H., Charifson, P.S., Bellon, S., Jernee, J., Dwyer, M., Chambers, S.P., Markland, W., Botfield, M., and Raybuck,

S.A. (2003). Active-site residues of Escherichia coli DNA gyrase required in coupling ATP hydrolysis to DNA supercoiling and amino acid substitutions leading to novobiocin resistance. Antimicrob. Agents Chemother. *47*, 1037–1046.

Guariglia-Oropeza, V., and Helmann, J.D. (2011). Bacillus subtilis σ(V) confers lysozyme resistance by activation of two cell wall modification pathways, peptidoglycan O-acetylation and D-alanylation of teichoic acids. J. Bacteriol. *193*, 6223–6232.

Hamperl, S., and Cimprich, K.A. (2016). Conflict Resolution in the Genome: How Transcription and Replication Make It Work. Cell *167*, 1455–1467.

Hamperl, S., Bocek, M.J., Saldivar, J.C., Swigut, T., and Cimprich, K.A. (2017). Transcription-Replication Conflict Orientation Modulates R-Loop Levels and Activates Distinct DNA Damage Responses. Cell *170*, 774–786.e19.

Hardy, C.D., and Cozzarelli, N.R. (2003). Alteration of Escherichia coli topoisomerase IV to novobiocin resistance. Antimicrob. Agents Chemother. *47*, 941–947.

Harwood, Colin, R., and Simon, M. Cutting. (1990). *Molecular biological methods for Bacillus* (Chichester: Wiley).

Heinz, S., Benner, C., Spann, N., Bertolino, E., Lin, Y.C., Laslo, P., Cheng, J.X., Murre, C., Singh, H., and Glass, C.K. (2010). Simple combinations of lineage-determining transcription factors prime cis-regulatory elements required for macrophage and B cell identities. Mol Cell *38*, 576–589, PMID: 20513432; PMCID: PMC2898526. https://doi.org/10.1016/j.molcel.2010.05.004.

Hiasa, H., and Marians, K.J. (1996). Two distinct modes of strand unlinking during theta-type DNA replication. J. Biol. Chem. *271*, 21529–21535.

Keszthelyi, A., Minchell, N.E., and Baxter, J. (2016). The Causes and Consequences of Topological Stress during DNA Replication. Genes (Basel) *7*, 134.

Khodursky, A.B., Peter, B.J., Schmid, M.B., DeRisi, J., Botstein, D., Brown, P.O., and Cozzarelli, N.R. (2000). Analysis of topoisomerase function in bacterial replication fork movement: use of DNA microarrays. Proc. Natl. Acad. Sci. USA *97*, 9419–9424.

Kuzminov, A. (2018). When DNA Topology Turns Deadly–RNA Polymerases Dig in Their R-loops to Stand Their Ground: New Positive and Negative (Super) Twists in the…. Trends Genet.

Lang, K.S., Hall, A.N., Merrikh, C.N., Ragheb, M., Tabakh, H., Pollock, A.J., Woodward, J.J., Dreifus, J.E., and Merrikh, H. (2017). Replication-Transcription Conflicts Generate R-Loops that Orchestrate Bacterial Stress Survival and Pathogenesis. Cell *170*, 787–799.e18.

Langmead, B., and Salzberg, S.L. (2012). Fast gapped-read alignment with Bowtie 2. Nat Methods *9*, 357–359, PMID: 22388286; PMCID: PMC3322381. https://doi.org/10.1038/nmeth.1923.

Levine, C., Hiasa, H., and Marians, K.J. (1998). DNA gyrase and topoisomerase IV: biochemical activities, physiological roles during chromosome replication, and drug sensitivities. Biochim. Biophys. Acta *1400*, 29–43.

Li, H., Handsaker, B., Wysoker, A., Fennell, T., Ruan, J., Homer, N., Marth, G., Abecasis, G., and Durbin, R.; 1000 Genome Project Data Processing Subgroup (2009). The Sequence Alignment/Map format and SAMtools. Bioinformatics *25*, 2078–2079.

Liu, L.F., and Wang, J.C. (1987). Supercoiling of the DNA template during transcription. Proc. Natl. Acad. Sci. USA *84*, 7024–7027.

Lodge, J.K., Kazic, T., and Berg, D.E. (1989). Formation of supercoiling domains in plasmid pBR322. J. Bacteriol. *171*, 2181–2187.

Lynch, A.S., and Wang, J.C. (1993). Anchoring of DNA to the bacterial cytoplasmic membrane through cotranscriptional synthesis of polypeptides encoding membrane proteins or proteins for export: a mechanism of plasmid hypernegative supercoiling in mutants deficient in DNA topoisomerase I. J. Bacteriol. *175*, 1645–1655.

Ma, J., Bai, L., and Wang, M.D. (2013). Transcription under torsion. Science *340*, 1580–1583.

Magner, D.B., Blankschien, M.D., Lee, J.A., Pennington, J.M., Lupski, J.R., and Rosenberg, S.M. (2007). RecQ promotes toxic recombination in cells lacking recombination intermediate-removal proteins. Mol. Cell *26*, 273–286.

Mangiameli, S.M., Merrikh, C.N., Wiggins, P.A., and Merrikh, H. (2017). Transcription leads to pervasive replisome instability in bacteria. eLife *6*, e19848.

Massé, E., and Drolet, M. (1999). Escherichia coli DNA topoisomerase I inhibits R-loop formation by relaxing transcription-induced negative supercoiling. J. Biol. Chem. *274*, 16659–16664.

Maxwell, A. (1993). The interaction between coumarin drugs and DNA gyrase. Mol. Microbiol. *9*, 681–686.

Merrikh, C.N., and Merrikh, H. (2018). Gene inversion potentiates bacterial evolvability and virulence. Nat. Commun. *9*, 4662.

Merrikh, H., Machón, C., Grainger, W.H., Grossman, A.D., and Soultanas, P. (2011). Co-directional replication-transcription conflicts lead to replication restart. Nature *470*, 554–557.

Merrikh, C.N., Brewer, B.J., and Merrikh, H. (2015). The B. subtilis Accessory Helicase PcrA Facilitates DNA Replication through Transcription Units. PLoS Genet. *11*, e1005289.

Million-Weaver, S., Samadpour, A.N., Moreno-Habel, D.A., Nugent, P., Brittnacher, M.J., Weiss, E., Hayden, H.S., Miller, S.I., Liachko, I., and Merrikh, H. (2015a). An underlying mechanism for the increased mutagenesis of lagging-strand genes in Bacillus subtilis. Proc. Natl. Acad. Sci. USA *112*, E1096–E1105.

Million-Weaver, S., Samadpour, A.N., and Merrikh, H. (2015b). Replication Restart after Replication-Transcription Conflicts Requires RecA in Bacillus subtilis. J. Bacteriol. *197*, 2374–2382.

Mirkin, E.V., and Mirkin, S.M. (2005). Mechanisms of transcription-replication collisions in bacteria. Mol. Cell. Biol. *25*, 888–895.

Mostertz, J., Scharf, C., Hecker, M., and Homuth, G. (2004). Transcriptome and proteome analysis of Bacillus subtilis gene expression in response to superoxide and peroxide stress. Microbiology (Reading) *150*, 497–512.

Nicolas, P., Mäder, U., Dervyn, E., Rochat, T., Leduc, A., Pigeonneau, N., Bidnenko, E., Marchadier, E., Hoebeke, M., Aymerich, S., et al. (2012). Condition-dependent transcriptome reveals high-level regulatory architecture in Bacillus subtilis. Science *335*, 1103–1106.

Nudler, E. (2009). RNA polymerase active center: the molecular engine of transcription. Annu. Rev. Biochem. *78*, 335–361.

Nudler, E. (2012). RNA polymerase backtracking in gene regulation and genome instability. Cell *149*, 1438–1445.

Ohtani, N., Haruki, M., Morikawa, M., Crouch, R.J., Itaya, M., and Kanaya, S. (1999). Identification of the genes encoding Mn2+-dependent RNase HII and Mg2+-dependent RNase HIII from Bacillus subtilis: classification of RNases H into three families. Biochemistry *38*, 605–618.

Paul, S., Million-Weaver, S., Chattopadhyay, S., Sokurenko, E., and Merrikh, H. (2013). Accelerated gene evolution through replication-transcription conflicts. Nature *495*, 512–515.

Peng, H., and Marians, K.J. (1993). Escherichia coli topoisomerase IV. Purification, characterization, subunit structure, and subunit interactions. J. Biol. Chem. *268*, 24481–24490.

Pomerantz, R.T., and O'Donnell, M. (2010). Direct restart of a replication fork stalled by a head-on RNA polymerase. Science *327*, 590–592.

Postow, L., Peter, B.J., and Cozzarelli, N.R. (1999). Knot what we thought before: the twisted story of replication. BioEssays *21*, 805–808.

Postow, L., Crisona, N.J., Peter, B.J., Hardy, C.D., and Cozzarelli, N.R. (2001a). Topological challenges to DNA replication: conformations at the fork. Proc. Natl. Acad. Sci. USA *98*, 8219–8226.

Postow, L., Ullsperger, C., Keller, R.W., Bustamante, C., Vologodskii, A.V., and Cozzarelli, N.R. (2001b). Positive torsional strain causes the formation of a four-way junction at replication forks. J. Biol. Chem. *276*, 2790–2796.

Prado, F., and Aguilera, A. (2005). Impairment of replication fork progression mediates RNA polII transcription-associated recombination. EMBO J. *24*, 1267–1276.

Promonet, A., Padioleau, I., Liu, Y., Sanz, L., Biernacka, A., Schmitz, A.-L., Skrzypczak, M., Sarrazin, A., Mettling, C., Rowicka, M., et al. (2020). Topoisomerase 1 prevents replication stress at R-loop-enriched transcription

CellPress
OPEN ACCESS

termination sites. Nat Commun *11*, 3940. https://doi.org/10.1038/s41467-020-17858-2.

Ramírez, F., Dündar, F., Diehl, S., Grüning, B.A., and Manke, T. (2014). deepTools: a flexible platform for exploring deep-sequencing data. Nucleic Acids Res. *42*, W187-91.

Randall, J.R., Hirst, W.G., and Simmons, L.A. (2018). Substrate Specificity for Bacterial RNases HII and HIII Is Influenced by Metal Availability. J. Bacteriol. *200*, 200.

Robinson, J.T., Thorvaldsdóttir, H., Winckler, W., Guttman, M., Lander, E.S., Getz, G., and Mesirov, J.P. (2011). Integrative genomics viewer. Nat. Biotechnol. *29*, 24–26.

Samadpour, A.N., and Merrikh, H. (2018). DNA gyrase activity regulates DnaA-dependent replication initiation in Bacillus subtilis. Mol Microbiol *108*, 115–127, Epub 2018 Mar 6. PMID: 29396913; PMCID: PMC5893406. https://doi.org/10.1111/mmi.13920.

Sanz, L.A., and Chédin, F. (2019). High-resolution, strand-specific R-loop mapping via S9.6-based DNA-RNA immunoprecipitation and high-throughput sequencing. Nat. Protoc. *14*, 1734–1755.

Schalbetter, S.A., Mansoubi, S., Chambers, A.L., Downs, J.A., and Baxter, J. (2015). Fork rotation and DNA precatenation are restricted during DNA replication to prevent chromosomal instability. Proc. Natl. Acad. Sci. USA *112*, E4565–E4570.

Smits, W.K., Goranov, A.I., and Grossman, A.D. (2010). Ordered association of helicase loader proteins with the Bacillus subtilis origin of replication in vivo. Mol. Microbiol. *75*, 452–461.

Smits, W.K., Merrikh, H., Bonilla, C.Y., and Grossman, A.D. (2011). Primosomal proteins DnaD and DnaB are recruited to chromosomal regions bound by DnaA in Bacillus subtilis. J. Bacteriol. *193*, 640–648.

Stolz, R., Sulthana, S., Hartono, S.R., Malig, M., Benham, C.J., and Chedin, F. (2019). Interplay between DNA sequence and negative superhelicity drives R-loop structures. Proc. Natl. Acad. Sci. USA *116*, 6260–6269.

Sugino, A., Higgins, N.P., Brown, P.O., Peebles, C.L., and Cozzarelli, N.R. (1978). Energy coupling in DNA gyrase and the mechanism of action of novobiocin. Proc. Natl. Acad. Sci. USA *75*, 4838–4842.

Tadesse, S., and Graumann, P.L. (2006). Differential and dynamic localization of topoisomerases in Bacillus subtilis. J. Bacteriol. *188*, 3002–3011.

Tuduri, S., Crabbé, L., Conti, C., Tourrière, H., Holtgreve-Grez, H., Jauch, A., Pantesco, V., De Vos, J., Thomas, A., Theillet, C., et al. (2009). Topoisomerase I suppresses genomic instability by preventing interference between replication and transcription. Nat. Cell Biol. *11*, 1315–1324.

Vos, S.M., Tretter, E.M., Schmidt, B.H., and Berger, J.M. (2011). All tangled up: how cells direct, manage and exploit topoisomerase function. Nat. Rev. Mol. Cell Biol. *12*, 827–841.

Wang, J.C. (2002). Cellular roles of DNA topoisomerases: a molecular perspective. Nat. Rev. Mol. Cell Biol. *3*, 430–440.

Wang, J.D., Berkmen, M.B., and Grossman, A.D. (2007). Genome-wide coorientation of replication and transcription reduces adverse effects on replication in Bacillus subtilis. Proc. Natl. Acad. Sci. USA *104*, 5608–5613.

Wu, H.Y., Shyy, S.H., Wang, J.C., and Liu, L.F. (1988). Transcription generates positively and negatively supercoiled domains in the template. Cell *53*, 433–440.

Zechiedrich, E.L., and Cozzarelli, N.R. (1995). Roles of topoisomerase IV and DNA gyrase in DNA unlinking during replication in Escherichia coli. Genes Dev. *9*, 2859–2869.

STAR★METHODS

KEY RESOURCES TABLE

REAGENT or RESOURCE	SOURCE	IDENTIFIER
**Antibodies**		
Mouse monoclonal S9.6 DNA:RNA Hybrid antibody	Millipore	MABE1095
Rabbit polyclonal anti-DnaC Antibody	Smits et al., 2010	N/A
Rabbit polyclonal anti-Gfp antibody	Merrikh et al., 2015	N/A
Mouse monoclonal anti-Myc antibody (clone 9E10)	Invitrogen	13-2500
Mouse monoclonal anti-RpoB antibody (clone 8RB13)	Thermo	MA125425
**Bacterial and virus strains**		
B. subtilis phe trp	Brehm et al., 1973	HM1
B. subtilis phe trp thrC::P_{xis}-*lacZ* (HO) ICEBs1(0)	Merrikh et al., 2015	HM211
B. subtilis phe trp thrC::P_{xis}-*lacZ* (HO)	Merrikh et al., 2015	HM640
B. subtilis phe trp amyE::$P_{spank(hy)}$-*lacZ* (HO)	Lang et al., 2017	HM1300
B. subtilis phe trp amyE::$P_{spank(hy)}$-*lacZ* (CD)	Lang et al., 2017	HM1416
B. subtilis phe trp thrC::P_{xis}-*lacZ* (HO) ICEBs1(0) *amyE*::$P_{spank(hy)}$-*sspB parC*::*parC*-ssrA	This study	HM1450
B. subtilis phe trp thrC::P_{xis}-*lacZ* (HO) *amyE*::$P_{spank(hy)}$-*sspB parC*::*parC*-ssrA	This study	HM1467
B. subtilis phe trp thrC::P_{xis}-*lacZ* (CD) *amyE*::$P_{spank(hy)}$-*sspB parC*::*parC*-ssrA	This study	HM1468
B. subtilis phe trp thrC::P_{xis}-*lacZ* (CD) ICEBs1(0) *amyE*::$P_{spank(hy)}$-*sspB parC*::*parC*-ssrA	This study	HM1469
B. subtilis phe trp thrC::P_{xis}-*lacZ* (CD) *amyE*::$P_{spank(hy)}$-*sspB gyrB*::*gyrB*-ssrA	This study	HM1949
B. subtilis phe trp thrC::P_{xis}-*lacZ* (CD) ICEBs1(0) *amyE*::$P_{spank(hy)}$-*sspB gyrB*::*gyrB*-ssrA	This study	HM1950
B. subtilis phe trp thrC::P_{xis}-*lacZ* (HO) *amyE*::$P_{spank(hy)}$-*sspB gyrB*::*gyrB*-ssrA	This study	HM1951
B. subtilis phe trp thrC::P_{xis}-*lacZ* (HO) ICEBs1(0) *amyE*::$P_{spank(hy)}$-*sspB gyrB*::*gyrB*-ssrA	This study	HM1952
B. subtilis phe trp amyE::$P_{spank(hy)}$-*lacZ* (HO) Δ*rnhC*::MLS	Lang et al., 2017	HM2043
B. subtilis phe trp amyE::$P_{spank(hy)}$-*lacZ* (CD) Δ*rnhC*::MLS	Lang et al., 2017	HM2044
B. subtilis phe trp thrC::P_{xis}-*lacZ* (HO) ICEBs1(0) *amyE*::$P_{spank(hy)}$-*sspB gyrB*::*gyrB*(R138L)-*ssrA*	This study	HM2420
B. subtilis phe trp thrC::P_{xis}-*lacZ* (CD) ICEBs1(0) *amyE*::$P_{spank(hy)}$-*sspB gyrB*::*gyrB*(R138L)-*ssrA*	This study	HM2421
B. subtilis phe trp thrC::P_{xis}-*lacZ* (HO) ICEBs1(0) *amyE*::$P_{spank(hy)}$-*sspB gyrB*::*gyrB*-myc-*ssrA*	This study	HM2442
B. subtilis phe trp thrC::P_{xis}-*lacZ* (HO) ICEBs1(0) *amyE*::$P_{spank(hy)}$-*sspB gyrB*::*parC*-myc-*ssrA*	This study	HM2444
B. subtilis phe trp Δ*rnhC*::MLS	Lang et al., 2017	HM2655
B. subtilis amyE::$P_{spank(hy)}$-*lacZ* (HO) *lacA*::$P_{spank(hy)}$-3x*myc-gfp*	This study	HM3019
B. subtilis amyE::$P_{spank(hy)}$-*lacZ* (CD) *lacA*::$P_{spank(hy)}$-3x*myc-gfp*	This study	HM3020
B. subtilis phe trp amyE::$P_{spank(hy)}$-*lacZ* (HO) *gyrA::gyrA-gfp*	This study	HM3863
B. subtilis phe trp amyE::$P_{spank(hy)}$-*lacZ* (CD) *gyrA::gyrA-gfp*	This study	HM3864
B. subtilis phe trp gyrB(R138L)	Samadpour and Merrikh, 2018	HM3387
B. subtilis phe trp Δ*rnhC*::MLS *gyrB*(R138L)	This study	HM4064

(*Continued on next page*)

Continued

REAGENT or RESOURCE	SOURCE	IDENTIFIER
B. subtilis phe trp Δ*rnhC*::MLS *gyrB*(R138L) *amyE*::$P_{spank(hy)}$-*lacZ* (HO)	This study	HM4065
B. subtilis phe trp Δ*rnhC*::MLS *gyrB*(R138L) *amyE*::$P_{spank(hy)}$-*lacZ* (CD)	This study	HM4066
B. subtilis phe trp amyE::$P_{spank(hy)}$-*lacZ* (HO) *parC*::*parC*-3xMyc	This study	HM4074
B. subtilis phe trp amyE::$P_{spank(hy)}$-*lacZ* (CD) *parC*::*parC*-3xMyc	This study	HM4075
Chemicals, peptides, and recombinant proteins		
EcoRV-HF	NEB	R0195
HindIII-HF	NEB	R0104
EcoRI-HF	NEB	R0101
DraI	NEB	R0129
RNase H	NEB	M0297
Protein A Sepharose	GE	GE17-0780-01
Critical commercial assays		
Nextera XT DNA Library Preparation Kit	Illumina	FC-131-1024
NEBNext DNA Library Prep master Mix Set	NEB	E6040
GeneJET PCR Purification Kit	Thermo	K0701
iScript supermix	Bio-Rad	1708840
iTaq Universal SYBR Green master mix	Bio-Rad	1725121
GeneJet Genomic DNA purication Kit	Thermo	K0721
Deposited data		
All sequencing DATA uploaded to the Sequence Read Archive	NCBI SRA	Bioproject PRJNA691533
Oligonucleotides		
GACATCCTCTGACAATCCTAGAG	This study	HM86
GGCAGTCACCTTAGAGTGCCCAAC	This study	HM87
GGCTTTCGCTACCTGGAGAG	Lang et al., 2017	HM188
GACGAAGCCGCCCTGTAAAC	Lang et al., 2017	HM189
CCGTCTGACCCGATCTTTTA	Lang et al., 2017	HM192
GTCATGCTGAATGTCGTGCT	Lang et al., 2017	HM193
AAGGCACATGGCTGAATATCG	Lang et al., 2017	HM910
ACACCAGACCAACTGGTAATGG	Lang et al., 2017	HM911
TTATGGATCCTGAAGGGTGAAGATGAACTG	This study	HM1690
TTATTCTAGATTGTTCTGTATGAAGGCGCCAAAC	This study	HM1691
ttatgaattcTATCGTAGAGGGTGACTCTG	This study	HM2282
TTATTCTAGAGATGTCAAGATTTTTAACGTATCTC	This study	HM2283
Recombinant DNA		
pGCS::*parC*	This study	pHM186
pGCS::*gyrB*	This study	pHM260
Software and algorithms		
SAMtools	Li et al., 2009	http://www.htslib.org/
Bowtie 2	Langmead and Salzberg, 2012	http://bowtie-bio.sourceforge.net/bowtie2/index.shtml
Prism 7	Graphpad	https://www.graphpad.com/scientific-software/prism/
DeepTools	Ramírez et al., 2014	https://deeptools.readthedocs.io/en/develop/
IGV	Robinson et al., 2011	http://software.broadinstitute.org/software/igv/
HOMER	Heinz et al., 2010	http://homer.ucsd.edu/homer/

RESOURCE AVAILABILITY

Lead contact

Further information and requests for resources and reagents should be directed to the Lead Contact, Houra Merrikh (houra.merrikh@vanderbilt.edu).

Materials availability

Materials generated in this study are available upon request.

Data and code availability

Datasets generated during this study are available from NCBI SRA project ID PRJNA691533.

EXPERIMENTAL MODEL AND SUBJECT DETAILS

Bacterial strains and growth conditions

Strains are listed in the key resources table. All strains were constructed in the HM1 (JH642) (Brehm et al., 1973) *B. subtilis* background. The *rnhC::mls* mutant (HM711) was obtained from the *Bacillus* genetic stock center (Columbus, OH). To move the *rnhC::mls* allele, genomic DNA was extracted from HM711 using a commercially available kit (Thermo) and used to transform into HM1 (and its derivatives with engineered conflict constructs) as per standard protocol (Harwood et al., 1990). Strains were streaked on LB agar plates and supplemented with antibiotics where appropriate. Precultures were inoculated from single colonies into 2 or 5 mL of LB broth and incubated at 37°C with shaking (260 RPM). Precultures were used to inoculate experimental cultures which were grown and treated as indicated for each different experiment in the materials and methods.

E. coli DH5α was used to propagate recombinant DNA vectors. Transformations were done using heat shock of competent *E. coli*. *E. coli* cultures were grown at 37°C with shaking (260 RPM) in LB supplemented with 50 μg/mL carbenicillin where appropriate. All plasmid vectors were purified using a commercially available plasmid extraction kit (Thermo).

Plasmid and strain constructions

pHM186 PCR was used to amplify 500 bp of the 3′ end of *parC* without the stop codon (primers HM1690/1691). The resulting amplicon was digested with BamHI and XbaI and ligated into pGCS (Griffith and Grossman, 2008).
pHM260 PCR was used to amplify 500 bp of the 3′ end of *gyrB* without the stop codon (primers HM22832284). The resulting amplicon was digested with EcoRI and XbaI and ligated into pGCS.
HM1450 Strain HM867 (Merrikh et al., 2015) was transformed with plasmid pHM186 and transformants were selected on LB plates containing chloramphenicol.
HM1467 Strain HM866 (Merrikh et al., 2015) was transformed with plasmid pHM186 and transformants were selected on LB plates containing chloramphenicol.
HM1468 Strain HM868 (Merrikh et al., 2015) was transformed with plasmid pHM186 and transformants were selected on LB plates containing chloramphenicol.
HM1469 Strain HM869 (Merrikh et al., 2015) was transformed with plasmid pHM186 and transformants were selected on LB plates containing chloramphenicol.
HM1949 Strain HM868 was transformed with plasmid pHM190 and transformants were selected on LB plates containing chloramphenicol.
HM1950 Strain HM869 was transformed with plasmid pHM190 and transformants were selected on LB plates containing chloramphenicol.
HM1951 Strain HM866 was transformed with plasmid pHM190 and transformants were selected on LB plates containing chloramphenicol.
HM1952 Strain HM867 was transformed with plasmid pHM190 and transformants were selected on LB plates containing chloramphenicol.
HM2420 Strain HM866 was transformed with genomic DNA purified from HM3387 and transformants were selected on LB plates containing novobiocin (4 μg/mL). The novobiocin resistant transformant was then transformed with pHM260.
HM2421 Strain HM869 was transformed with genomic DNA purified from HM3387 and transformants were selected on LB plates containing novobiocin (4 μg/mL). The novobiocin resistant transformant was then transformed with pHM260.
HM4064 Strain HM3387 was transformed with gDNA purified from strain HM2655 and transformants were selected for on LB containing erythromycin and lincomycin.
HM4065 Strain HM4064 was transformed with plasmid pHM171 (Lang et al., 2017).
HM4066 Strain HM4064 was transformed with plasmid pHM180 (Lang et al., 2017).

METHOD DETAILS

Viability assays

Strains were struck on LB plates supplemented with the appropriate antibiotic from freezer stocks and incubated overnight at 37°C. Single colonies were used to inoculate 2 mL LB cultures in glass tubes. The cultures were grown at 37°C with shaking (260 RPM) to OD600 = 0.5-1.0. Precultures were adjusted to OD 0.3 and then serially diluted in 1x Spizzen's Salts (15 mM ammonium sulfate, 80 mM dibasic potassium phosphate, 44 mM monobasic potassium phosphate, 3.4 mM trisodium citrate, and 0.8 mM magnesium sulfate). 5ul of each dilution was plated onto LB plates and incubated at 30°C overnight. For survival assays with the engineered conflict strains, LB plates were either supplemented or not with various concentrations of novobiocin and/or IPTG as indicated in the figure legends. For the type II topoisomerase degron experiments, chloramphenicol was added to the media to maintain the stability of degron tag. For chronic cell wall stress assays, LB plates were supplemented with lysozyme to a final concentration of 50 μg/mL. Plates were imaged with a BioRad Gel Doc™ XR+ Molecular Imager® and colonies were enumerated.

Slot blot analysis

Precultures grown from single colonies were diluted back to OD 0.05 in replicate cultures and grown until OD600 = 0.3. IPTG was added to one replicate of each strain to a final concentration of 0.1 mM. For each strain, 3 mL of culture was spun at 10k RPM for 3 min and washed with 1x PBS. Cell pellets were resuspended in 300 μL Lysis Buffer (TE pH 8.0, 0.1 mg/mL lysozyme, and 1x AEBSF) and incubated at 37°C for 30 min. Cells were lysed by the addition of SDS to a final concentration of 1%. Samples were then boiled for 10 minutes. Total protein levels were measured using a Qubit protein quantification assay. 40 μg of each sample was applied to a PVDF membrane via a Slot Blot apparatus (Bio-Rad). Membrane were then blocked in Odyssey Buffer, and anti-myc antibody (910E1, invitrogen) was added (1:500) for overnight incubation at 4°C. Membranes were washed 5x in PBST. Membranes were then incubated with an anti-mouse Odyssey secondary antibody (1:15,000 in Odyssey Buffer) for 30 mins. Membranes were then washed 3x in PBST and imaged on a Li-Cor Imager.

Chromatin immunoprecipitation assays (ChIPs)

Precultures were diluted to OD600 of 0.05 in LB and grown at 30°C with shaking. At OD600 ~0.1, cultures were induced with 1 mM IPTG (final concentration) and grown until the culture was at OD600 = 0.3 and processed as described (Merrikh et al., 2011). Briefly, cultures were crosslinked with 1% formaldehyde or ciprofloxacin (4 ug/mL, Topo IV only) for 20 minutes and subsequently quenched with 0.5 M glycine (formaldehyde crosslinking only). Cell pellets were collected by centrifugation and washed once with cold phosphate buffered saline (PBS). Cell pellets were resuspended with 1.5 mL of Solution A (10 mM Tris–HCl pH 8.0, 20% w/v sucrose, 50 mM NaCl, 10 mM EDTA, 10 mg/ml lysozyme, 1 mM AEBSF) and incubated at 37°C for 30 min. After incubation, 1.5 mL of 2x IP buffer (100 mM Tris pH 7.0, 10 mM EDTA, 20% triton x-100, 300 mM NaCl and 1mM AEBSF) was added and lysates were incubated on ice for 30 minutes. Lysates were then sonicated 4 times at 30% amplitude for 10 s of sonication and 10 s of rest. Lysates were pelleted by centrifugation at 8000 RPMs for 15 minutes at 4°C. Each IP was done with 1 mL of cell lysate and 40 μL was taken out prior to addition of the antibody as an input control. IPs were performed using rabbit polyclonal antibodies against DnaC (Smits et al., 2010), RNAP (Santa Cruz Biotech), GFP (Abcam, gyrase) and Myc (Invitrogen, Topo IV). IPs were rotated overnight at 4°C. After incubation with the antibody, 30 μL of 50% Protein A Sepharose beads (GE) were added and IPs were incubated at RT for one hour with gentle rotation. Beads were then pelleted by centrifugation at 2000 RPM for 1 minute. The supernatant was removed and the beads were washed 6x with 1mL of 1x IP buffer. An additional wash was done with 1 mL of TE pH 8.0. After the washes, 100 μL of elution buffer I (50 mM Tris pH 8.0, 10 mM EDTA, 1% SDS) was added and beads were incubated at 65°C for 10 minutes. Beads were pelleted by centrifugation at 5000 RPMs for 1 minute. The supernatant was removed, saved and 150 μL of elution buffer II (10 mM Tris pH 8.0, 1 mM EDTA, 0.67% SDS) was added. Beads were then pelleted by centrifugation at 7000 RPMs for 1 minute and the supernatant was combined with the first elution. The combined eluates were then de-crosslinked by incubation at 65°C for overnight. The eluates were then treated with proteinase K (0.4 mg/mL) at 37°C for 2 hours. DNA was then extracted with a GeneJet PCR purification Kit (Thermo) according to the manufacturer's instructions or a standard phenol:chloroform extraction.

DNA:RNA hybrid immunoprecipitation assays (DRIPs)

DRIPs were performed as described with modifications for use in bacteria(García-Rubio et al., 2018; Lang et al., 2017; Sanz and Chédin, 2019). Precultures were diluted to OD600 of 0.05 in LB and grown at 30°C with shaking. At OD600 ~0.1, cultures were induced with 1 mM IPTG (final concentration) and grown until the culture was at OD600 = 0.3. Cells were pelleted by centrifugation and washed twice with cold PBS. Total nucleic acids were purified from cell pellets using phenol:chloroform extraction and ethanol precipitation. Precipitated DNA was spooled on a glass rod and after drying, DNA was resuspended in TE pH 8.0 and treated with EcoRV, EcoRI, DraI, and HindIII overnight at 37°C. Cutsites in the engineered locus (and ~20 kb window surrounding) are listed in Table S4. Digested chromosomal DNA was then purified by phenol:chloroform extraction and brought to final volume of 125 μL. For the RNase H treated controls, 10 μg was treated with 3 μL of RNase H (NEB) in 1x RNase H buffer at 37°C overnight prior to immunoprecipitation. Nucleic acids were then quantified using a Qubit (Invitrogen) and 10 μg were added to each IP in 500 total μL of TE. 50 μL was then removed kept as INPUT. 52 μL of 10x Binding buffer (100 mM $NaPO_4$ pH 7.0, 1.4 M NaCl, 0.5% Triton X-100) was added. S9.6 antibody (Millipore) was added (20 μL) and samples were incubated overnight at 4°C with gentle rotation. After incubation with

the antibody, 40 μL of 50% Protein A Sepharose beads (GE) were added and IPs were incubated at 4°C for 2 hours with gentle rotation. Beads were then pelleted by centrifugation at 2000 RPM for 1 minute. The supernatant was removed and the beads were washed 3x with 1mL of 1x Binding buffer. After the washes, 300 μL of elution buffer (10 mM Tris pH 8.0, 1 mM EDTA, 0.67% SDS) and 7 μL Proteinase K (QIAGEN) were added. For the INPUT samples, 3 μL Proteinase K was added. All samples were incubated at 55°C for 45 minutes with gentle rotation. Beads were then pelleted by centrifugation at 7000 RPMs for 1 minute and the supernatant moved to a new tube. DNA was purified by phenol:chloroform extraction and ethanol precipitation and used to prepare Illumina libraries using the Nextera NT library prep kit (Illumina) or the NEBNext Library Prep Kit (NEB) or analyzed using qPCR. DRIP-qPCR analysis was done by the ratio of signal at the conflict region (primer pair 188/189 or 910/911) divided by a control locus *yhaX* (192/193).

RNA extraction and cDNA preparation

Cells were grown in LB to mid-exponential phase and back diluted to OD600 0.05 into LB either supplemented with or lacking 1mM IPTG. Cells were grown for 2 hours at 30°C (3 generations) prior to harvesting. 5 mL of culture was harvested by addition to an equal volume of ice-cold methanol followed by centrifugation at 4,000xg for 5 minutes. Cells were lysed with 20 μg/mL lysozyme for 10 minutes. RNA was isolated with the GeneJET RNA Purification Kit (ThermoFisher Scientific). 1 μg of RNA was treated with RNase-free DNase I (ThermoFisher Scientific) for 40 minutes at 37°C. DNase I was denatured by the addition of 1ul of EDTA and incubation at 65°C for 10 minutes. Reverse transcription was performed with iScript Supermix (BioRad) as per manufacturer's instructions. mRNA abundance was measured via qPCR analysis by measuring the signal ratio of the target locus *lacZ* (primer pair 188/189) by the control *rrn* locus (primer pair 86/87).

Next generation sequence analysis

Sequencing libraries were generated using either the Nextera NT library prep kit from Illumina or by standard end polishing and ligation with the NEBNext adaptor kit (NEB). Approximately 4M x 150 bp paired-end or single-end Illumina Next-Seq reads per sample were quality and adaptor trimmed using Trimmomatic (Bolger et al., 2014) and mapped to the genome of *B. subtilis* strains HM1300 (head-on lacZ) and HM1416 (co-directional lacZ) in the strain background JH642 (GenBank: CP007800.1) using Bowtie 2 (Langmead and Salzberg, 2012). Bam files were normalized for the total number of mapped reads and the ratio of the immunoprecipitation versus the input was done using the deepTools bamCompare tool (Ramírez et al., 2014). Plots were generated in IGV (Robinson et al., 2011). Peaks were identified and analyzed using the HOMER (Heinz et al., 2010) findPeaks tool, analyzing the mapped read files for the IP compared to the input. Read density in the identified peak regions was quantified using HOMER's annotatePeaks function using the normalized bedgraph files (IP/input) generated from deepTools.

QUANTIFICATION AND STATISTICAL ANALYSIS

Analysis of deep sequencing data was done using HOMER as described in Method details. Statistical analysis of ChIP-qPCR and RT-qPCR data was done in Prism 8 as described in Method details.

Cell Reports, Volume *34*

Supplemental information

Topological stress is responsible for the detrimental outcomes of head-on replication-transcription conflicts

Kevin S. Lang and Houra Merrikh

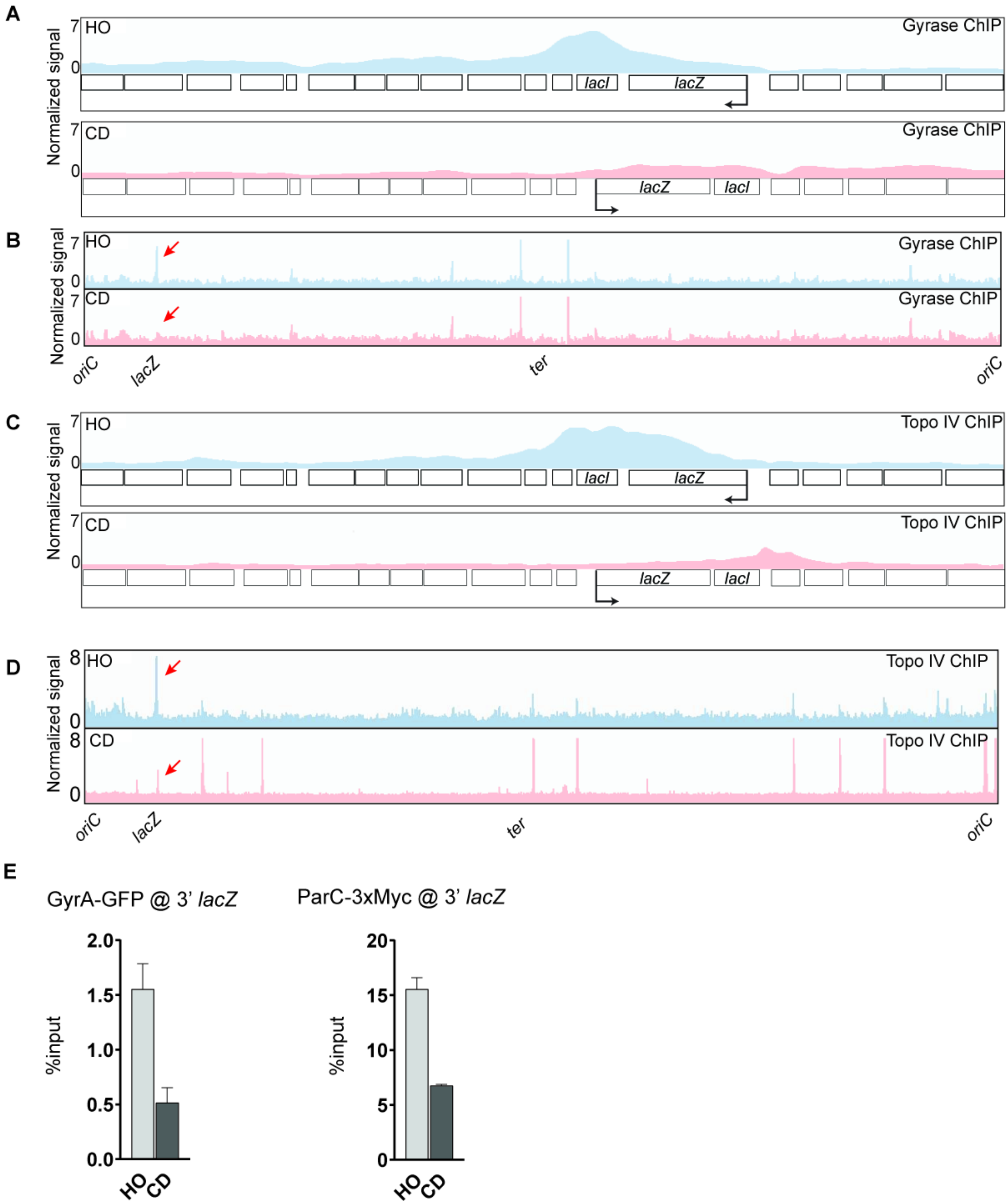

Supplementary Figure 1. Association of type II topoisomerases with head-on genes is dependent on transcription. Related to Figure 1. Replicate ChIP-Seq profiles of **(A)** gyrase and **(C)** Topo IV at the engineered conflict locus and **(B)** and **(D)** genome-wide in cells carrying either a head-on (HO, blue, strain HM3863 (gyrase), HM4074 (ParC)) or co-directional (CD, red, strain HM3864 (gyrase), HM4075 (ParC)) *lacZ* engineered conflict. The direction of DNA replication is left to right. Direction of transcription is indicated by the promoter arrow on *lacZ*. **(E)** ChIP-qPCR data from **Fig. 1C-D** presented as percent input. Bars represent the mean and standard error.

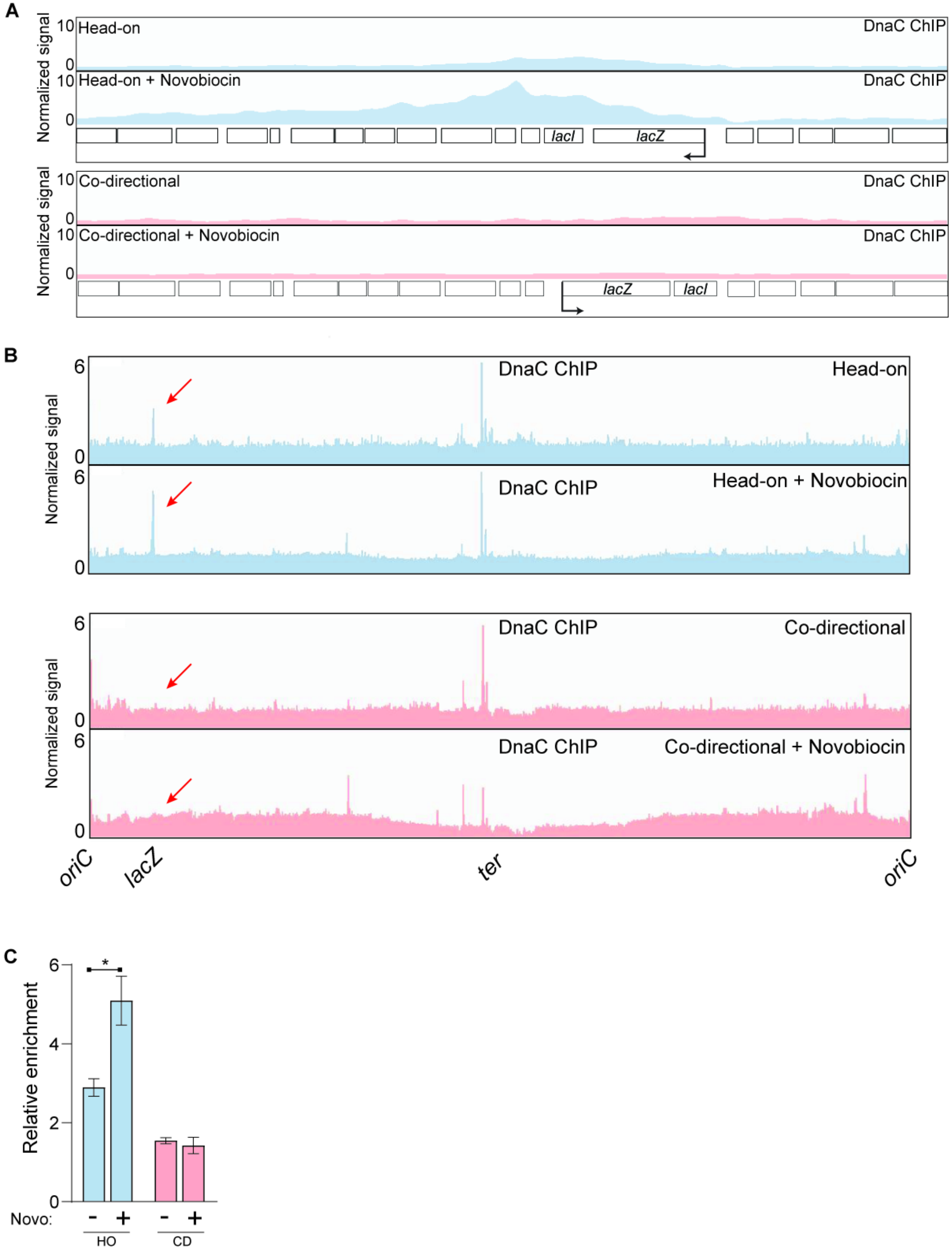

Supplementary Figure 2. Association of the replicative helicase at engineered conflict regions. Related to Figure 2. Replicate ChIP-Seq profiles at the engineered conflict locus **(A)** and genome-wide **b,** of DnaC in cells carrying either a head-on (HO, blue, strain HM1300) or co-directional (CD, red, strain HM1416) *lacZ*

engineered conflict, with and without novobiocin treatment (375 ng/mL). The direction of DNA replication is left to right. Direction of transcription is indicated by the promoter arrow on *lacZ*. Red arrow shows the location of the engineered conflict locus in the genome-wide plot. **c,** ChIP-qPCR analysis of the 3' region of *lacZ* with and without novobiocin treatment. The bars represent the mean and standard error of 4 biological replicates. (*) represents $p < 0.05$.

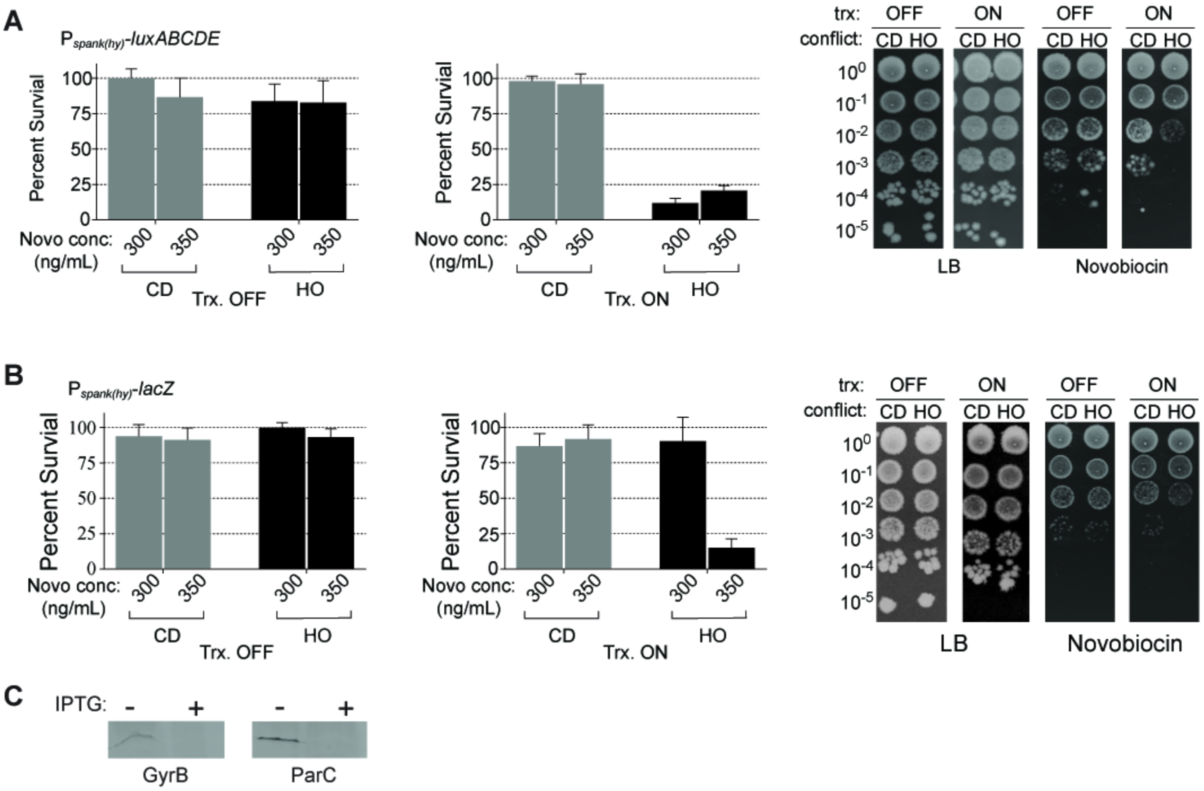

Supplementary Figure 3. Sensitivity of cells expressing a highly transcribed gene is independent of gene sequence and genomic location. Related to Figure 3. Novobiocin survival assays of cells expressing $P_{spank(hy)}$-*luxABCDE* **(A)** and $P_{spank(hy)}$-*lacZ* **(B)** at the *amyE* locus. Quantification is shown as percent survival (average, +/- standard deviation). Representative plates of the highest novobiocin concentration (350 ng/mL) are shown. **(C)** Slot blot analysis of depletions of GyrB and ParC using the degron systems.

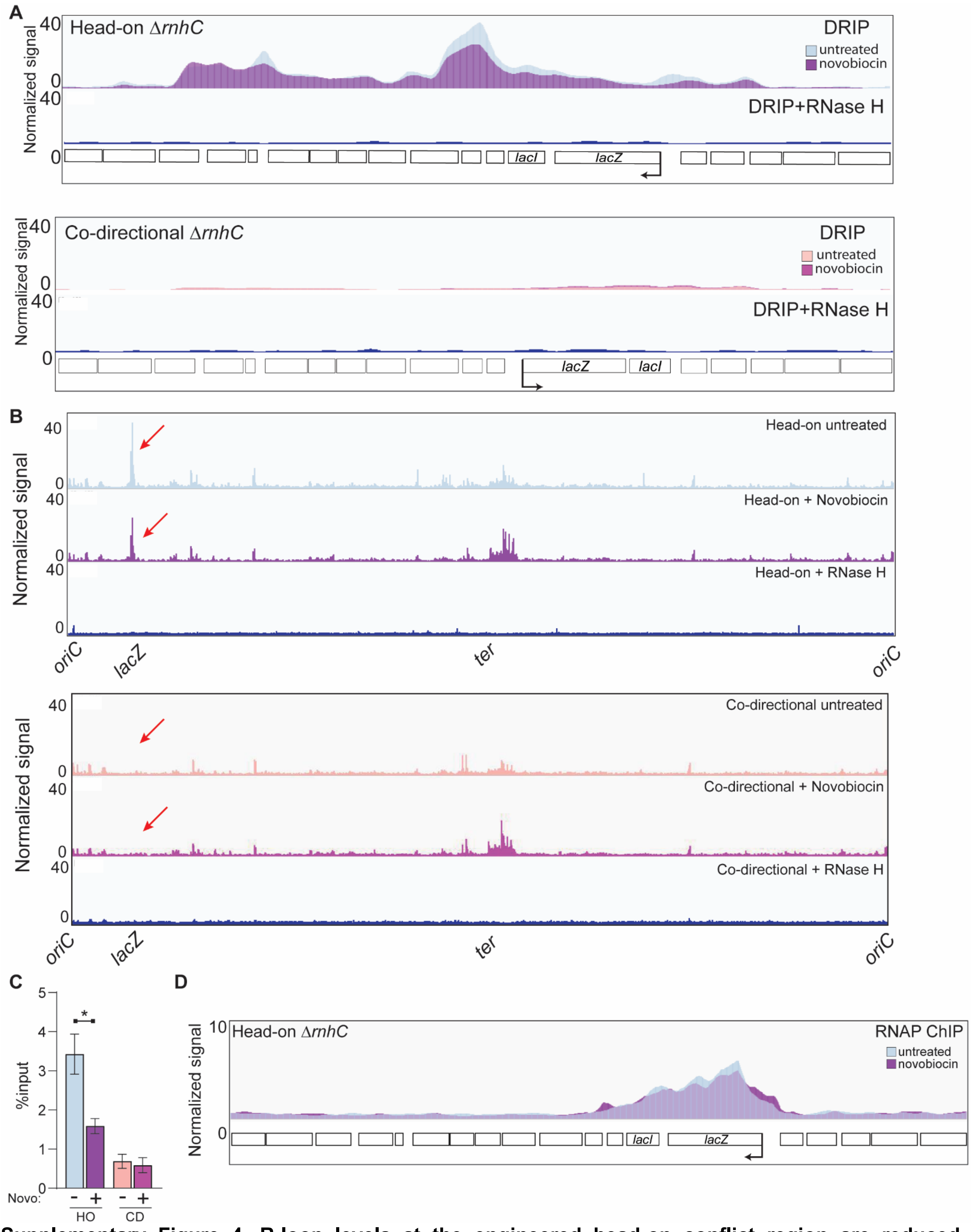

Supplementary Figure 4. R-loop levels at the engineered head-on conflict region are reduced in novobiocin treated cells. Related to Figure 4 (A) Replicate DRIP-Seq profiles of cells lacking RNase HIII harboring either a head-on (HO, blue, strain HM2043) or co-directional (CD, red, strain HM2044) *lacZ* engineered

conflict treated or untreated with novobiocin. Genome-wide maps are shown in **(B).** The bottom panel of all DRIP-Seq plots are the RNase H treated controls. **(C)** DRIP-qPCR analysis of the 3' region of *lacZ* with and without novobiocin treatment represented as percent input. The bars represent the mean and standard error of 4 biological replicates. (*) represents $p < 0.05$. **(D)** Representative RNAP ChIP-seq plot of Δ*rnhC* cells expressing the head-on engineered conflict untreated (blue) and treated with novobiocin (magenta). The conditions are shown as an overlay.

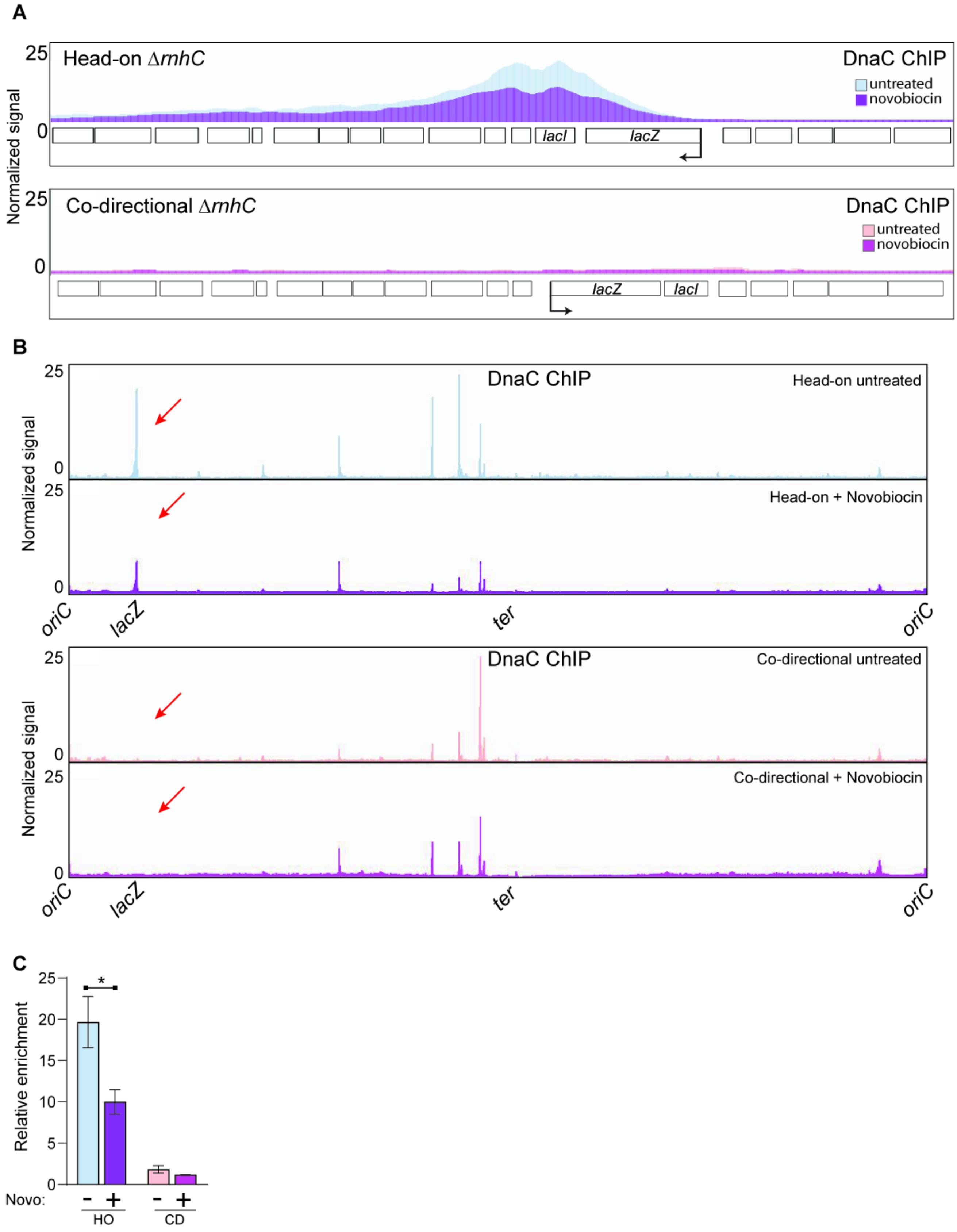

Supplementary Figure 5. Replication fork stalling due to R-loops at the engineered head-on conflict region is reduced with type II topoisomerase inhibition. Related to Figure 4. (A) Replicate DnaC ChIP-Seq profiles of cells lacking RNase HIII harboring either a head-on (HO, blue, strain HM2043) or co-directional (CD, red, strain HM2044) *lacZ* engineered conflict treated or untreated with novobiocin. Genome-wide maps are shown in **(B)**. **(C)** ChIP-qPCR analysis of the 3' region of *lacZ* with and without novobiocin treatment in cells lacking *rnhC*. The bars represent the mean and standard error of 4 biological replicates. (*) represents $p < 0.05$.

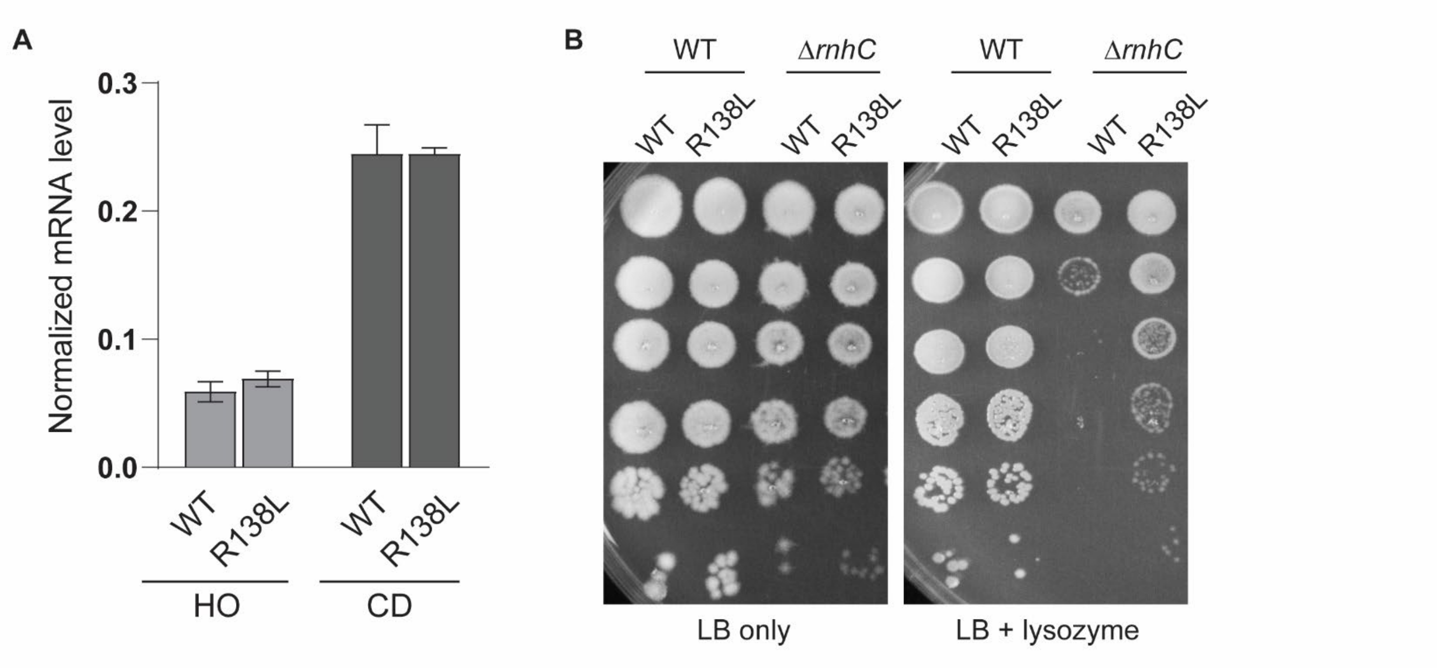

Supplementary Figure 6. Transcript levels of *lacZ* do not change in the R138L gyrase mutant, but stress response defects are rescued. Related to Figure 5. (A) qRT-PCR analysis measuring mRNA levels of *lacZ* relative to a housekeeping gene *rrs* in cells lacking RNase HIII with either the WT or R138L *gyrB* allele harboring either a head-on (HO, HM2043/HM4065) or co-directional (CD, HM2044/HM4066) *lacZ* engineered conflict. **(B)** Representative survival assays of WT cells or cells lacking RNase HIII with either the WT or R138L *gyrB* allele plated on either LB or LB containing 50 μg/ml of lysozyme.

Supplementary Table 1. Gyrase ChIP-Seq peaks identified genome-wide. Related to Figure 1.

start[a]	end[a]	peak score[b]	nearest gene
2197501	2199971	55457.4	yonT
1981403	1984331	42368.6	yoeD
327538	329328	33419.7	lacZ-lacI
3755421	3755631	3346.4	ywmC
1671341	1671441	720.7	sucD

a. Start and end genomic coordinates of peaks
b. Peak score calculated by Homer (IP compared to input)

Supplementary Table 2. ParC ChIP-Seq peaks identified genome-wide. Related to Figure 1.

start[a]	end[a]	peak score[b]	nearest gene
326665	331715	110502	*lacZ-lacI*
4189638	4190485	18922.1	*trmF*
5273	6189	17170.5	*gyrB*
4190470	4191199	15568.1	*trmE*
4187156	4187531	4796	*noc*
138158	138458	4278.9	*rpsS*
3935623	3936099	3978.1	*ilvK*
326461	326761	3445.6	*ycgB*
3936882	3937182	2945.5	*ilvK*
4191433	4191847	2442.2	*trmE*
4152542	4152992	1863.4	*yybL*
325129	325429	1773.5	*ycgA*
4153041	4153501	1677	*yybL*
4110512	4110890	1352.8	*yydD*
4186262	4186562	1217.4	*yyaB*
3646262	3646562	1010.7	*ggaB*
4153729	4154029	911.9	*yybK*

a. Start and end genomic coordinates of peaks
b. Peak score calculated by Homer (IP compared to input)

Supplementary Table 3. DnaC ChIP-Seq peaks identified genome-wide in wild-type cells. Related to Figure 2.

start[a]	end[a]	peak score[b]	nearest gene	average signal intensity (+/- std dev)[c]	
				untreated	treated
323023	331140	174468	*lacZ-lacI*	1.939(+/-0.073)	4.297(+/-1.11)
243	3049	41350.5	*dnaN*	3.3675(+/-0.415)	3.198(+/-1.075)
3936777	3937154	5900.9	*licH*	1.222(+/-0.094)	2.8335(+/-0.929)
1997029	1997908	4823.2	*yoxB*	15.537(+/-0.357)	6.4325(+/-2.001)
4193215	4193743	4189.2	*oxaAA*	2.387(+/-0.297)	2.518(+/-0.961)
3935747	3936047	2306.7	*ilvK*	1.394(+/-0.087)	2.495(+/-0.456)
1763564	1763864	1175.3	*cotE*	1.306(+/-0.058)	1.743(+/-0.673)

a. Start and end genomic coordinates of peaks
b. Peak score calculated by Homer (IP compared to input)
c. Average read density (ip/input) across replicate experiments within the peak regions.

Supplementary Table 4. Cut sites used for DRIP-Seq chromosome fragmentation at the engineered conflict locus. Related to Figure 4.

Fragment length	Enzyme sites
2076	EcoRI-5997-DraI-8073
1963	EcoRI-14898-EcoRV-16861
1481	DraI-19469-DraI-20950
1219	DraI-8753-EcoRV-9972
1178	EcoRV-16861-HindIII-18039
1074	HindIII-3686-DraI-4760
987	EcoRV-13788-DraI-14775
831	DraI-4760-DraI-5591
822	HindIII-2534-HindIII-3356
797	HindIII-353-HindIII-1150
779	HindIII-24016-HindIII-24795
675	DraI-18794-DraI-19469
663	DraI-8073-HindIII-8736
663	HindIII-11589-DraI-12252
657	DraI-23340-EcoRV-23997
624	DraI-22242-DraI-22866
599	DraI-13189-EcoRV-13788
551	EcoRV-21179-DraI-21730
512	DraI-21730-DraI-22242
490	DraI-12699-DraI-13189
474	DraI-22866-DraI-23340
451	DraI-2083-HindIII-2534
447	DraI-12252-DraI-12699
424	EcoRI-10979-HindIII-11403
395	HindIII-18039-EcoRI-18434
381	DraI-10598-EcoRI-10979
353	start-HindIII-353
307	EcoRV-9972-HindIII-10279
291	EcoRV-1792-DraI-2083
273	HindIII-3413-HindIII-3686
263	DraI-18531-DraI-18794
237	HindIII-1150-DraI-1387
229	DraI-20950-EcoRV-21179
211	HindIII-10387-DraI-10598
208	EcoRV-25043-end
186	HindIII-11403-HindIII-11589
177	EcoRV-1615-EcoRV-1792
175	HindIII-24868-EcoRV-25043
162	DraI-1387-EcoRV-1549
155	DraI-5591-DraI-5746
126	DraI-5746-DraI-5872
125	DraI-5872-EcoRI-5997
123	DraI-14775-EcoRI-14898
108	HindIII-10279-HindIII-10387
97	EcoRI-18434-DraI-18531
73	HindIII-24795-HindIII-24868

66	EcoRV-1549-EcoRV-1615
57	HindIII-3356-HindIII-3413
19	EcoRV-23997-HindIII-24016
17	HindIII-8736-DraI-8753

Supplementary Table 5. DRIP-Seq peaks identified genome-wide in Δ*rnhC* cells. Related to Figure 4.

start[a]	end[a]	peak score[b]	nearest gene	average signal intensity (+/- std dev)[c]	
				untreated	treated
326514	328288	104923.5	*lacZ-lacI*	31.8915(+/-3.2885)	23.727(+/-2.849)
320595	321068	10352.6	*yceK*	21.464(+/-2.932)	11.642(+/-2.876)
2195721	2196840	9727.1	*yonX*	17.494(+/-5.43)	13.774(+/-3.09)
2205720	2206116	4312.5	*yonK*	6.863(+/-0.655)	12.199(+/-1.829)
2199715	2200167	3476	*yonR*	15.2025(+/-5.6295)	9.519(+/-2.333)
3156556	3156856	3125.6	*thiT*	4.044(+/-0.814)	4.1605(+/-1.4335)
2195342	2195728	2761.9	*yopA*	15.323(+/-5.207)	14.4445(+/-3.7375)
2203688	2203988	2586.7	*yonN*	5.282(+/-0.317)	8.936(+/-0.976)
942449	942749	2149.6	*spo0M*	11.3975(+/-0.0865)	7.1765(+/-0.7225)
1894389	1894764	2115.7	*cotC*	8.957(+/-1.865)	3.2955(+/-0.4705)
2199497	2199797	1706.9	*yonS*	14.1335(+/-5.7965)	10.0885(+/-2.0395)
2225847	2226332	1652.3	*youB*	8.043(+/-1.796)	8.5895(+/-2.7065)
2200908	2201208	1612.7	*yonO*	11.68(+/-3.27)	8.126(+/-1.701)
2165020	2165320	1053.2	*yorF*	4.725(+/-0.678)	3.674(+/-0.432)
2184825	2185125	1011.7	*yopQ*	6.2645(+/-1.7985)	4.491(+/-1.051)
100138	100438	906.2	*ctsR*	1.714(+/-0.265)	1.7595(+/-0.4875)

a. Start and end genomic coordinates of peaks
b. Peak score calculated by Homer (IP compared to input)
c. Average read density (ip/input) across replicate experiments within the peak regions.

Supplementary Table 6. DnaC ChIP-Seq peaks identified genome-wide in Δ*rnhC* cells. Related to Figure 4.

start[a]	end[a]	peak score[b]	nearest gene	average signal intensity (+/- std dev)[c]	
				untreated	treated
318699	331480	325183.6	*lacZ-lacI*	12.169(+/-2.378)	7.8815(+/-1.3485)
1892711	1896089	50375	*cotC*	16.607(+/-4.462)	4.157(+/-0.433)
1995150	1998713	49744.2	*rtp*	11.6215(+/-1.5545)	6.8645(+/-0.5535)
1762517	1765736	46372.4	*cotE*	12.973(+/-2.762)	3.7645(+/-1.4635)
1311141	1312741	23008.7	*xkdC*	9.7685(+/-4.1445)	8.5015(+/-1.2095)
317781	318312	10485.9	*niaP*	4.736(+/-1.022)	3.743(+/-0.756)
317248	317513	4803.1	*niaP*	4.5265(+/-1.3085)	3.2575(+/-0.5705)
316756	316980	2770	*yceH*	4.2335(+/-1.4415)	2.786(+/-0.585)
2014811	2015077	1986.8	*yoaM*	4.3525(+/-0.6565)	3.5695(+/-0.5045)
2015730	2016078	1863.4	*yozS*	5.3065(+/-0.4825)	3.3(+/-1.395)
942138	942288	983.8	*spo0M*	4.2355(+/-1.2235)	1.812(+/-0.072)
1892228	1892378	513.1	*thyA*	3.5535(+/-0.1195)	1.0505(+/-0.2205)
941681	941831	499.6	*spo0M*	3.579(+/-0.523)	1.777(+/-0.256)
1896289	1896439	486.1	*ynzB*	3.802(+/-0.718)	1.5965(+/-0.1455)

a. Start and end genomic coordinates of peaks
b. Peak score calculated by Homer (IP compared to input)
c. Average read density (ip/input) across replicate experiments within the peak regions.
